# Drop‐Shaped Optical Microfiber Enabled Biomechanical Sensor

**DOI:** 10.1002/advs.75673

**Published:** 2026-05-14

**Authors:** Yan Xu, Xitao Tu, Haochen Jiang, Jun Mo, Xinyue Zhang, Xiaomin Yue, Limin Tong, Lei Zhang

**Affiliations:** ^1^ State Key Laboratory of Extreme Photonics and Instrumentation College of Optical Science and Engineering Zhejiang University Hangzhou China; ^2^ Department of Biophysics and Department of Neurosurgery of the Fourth Affiliated Hospital Zhejiang University School of Medicine Hangzhou China; ^3^ Department of Neurosurgery The Fourth Affiliated Hospital of School of Medicine and International School of Medicine International Institutes of Medicine Zhejiang University Yiwu China

**Keywords:** biomechanics, C. elegans, microforce sensor, optical microfiber, single cell, Young's modulus

## Abstract

Microforce sensing plays a pivotal role in biomechanics. However, existing microforce sensors face limitations in conducting biomechanical sensing on living organisms due to cumbersome operational procedures and limited measurement flexibility. To overcome this challenge, we developed a microforce sensor using a drop‐shaped optical microfiber. Leveraging its small bending radius and the bending‐radius‐dependent output light intensity, the microforce sensor features a small footprint, a low spring constant, and enables nanonewton‐level force sensing by monitoring variations in output light intensity. To prevent contact induced surface contamination, a PDMS microsphere is integrated at the sensor tip to isolate the waveguide evanescent field and boost microforce detection sensitivity. The sensor exhibits a force resolution of 24 nN in the range of 0–10 µN. As proof‐of‐concept demonstrations, Young's modulus characterization of onion epidermal cells and *Caenorhabditis elegans* are realized. The sensor demonstrated here is promising to offer a precise, flexible, and reliable solution for biomechanical characterization and is configurable to cater to a broad spectrum of applications.

## Graphical abstract

A novel microforce sensor based on a drop‐shaped optical microfiber is presented herein, designed to characterize the biomechanical properties of single cells and microscale living organisms, achieving a force resolution of 24 nN. Leveraging its high sensitivity, facile miniaturization, excellent flexibility, and robust mechanical stability, this sensor exhibits considerable potential for broad applications across biomechanics, cell mechanics, and materials science.

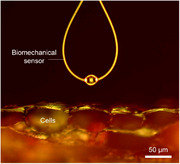

## 1 Introduction

Precise measurement of microforce plays a crucial role in biomechanics [1, 2], materials science [3, 4], molecular biology [5], and micromanipulation [6, 7]. For instance, measuring the biomechanical properties of single cells or tissues (e.g., Young's modulus, adhesion force, etc.) provides multidimensional insights for life sciences, physiology, and medical research, enhancing our understanding of life processes, physiological mechanisms, and pathological developments [1, 2, 8, 9, 10]. Currently, biomechanical research primarily relies on advanced techniques, such as atomic force microscopy (AFM) [11, 12, 13, 14], optical tweezers [15, 16, 17], and magnetic tweezers [18, 19, 20]. However, they often face limitations in conducting in situ biomechanical sensing on single cells or living organisms due to complex operational procedures and limited measurement flexibility. To address these challenges, researchers have developed a wide range of novel optical [21, 22, 23], electrical [24], or magnetic [25] microforce sensors for characterizing the biomechanical properties of cells, tissues, and other biological specimens [26].

Among various microforce sensing platform, optical fiber‐based sensors have attracted considerable interest owing to their high sensitivity, fast response, and miniature size. For example, Elwenspoek et al. fabricated a Fabry‐Pérot (FP) cavity by engraving a microcantilever on the end face of a single‐mode fiber, serving as a viable alternative to conventional AFMs [27], Recently, various of fiber tip FP sensors have been fabricated via two‐photon polymerization nanolithography [28], which highlights their potential for biomechanical sensing [29, 30]. Compared to standard optical fibers, optical microfibers possess ultralow optical propagation loss, strong evanescent field, a micrometer‐scale bending radius, and a gigapascal (GPa)‐level tensile strength [31, 32, 33, 34]. Owing to these distinctive optical and mechanical properties, optical microfibers have attracted considerable attention in tactile sensing [35, 36, 37], biochemical analysis [38, 39, 40, 41], ultrasonic imaging [42, 43] and microforce sensing [44, 45, 46, 47]. Unlike AFM cantilevers, which rely on complicated optical systems to detect cantilever's displacement, U‐shaped optical microfiber cantilevers exhibit ultralow spring constants in the range of 0.15mN m^−1^–2N m^−1^, together with self‐sensing capability, rendering them highly promising candidates for microforce sensing applications [45, 46].

Here, we report a microforce sensor using a drop‐shaped optical microfiber with a PDMS microsphere integrated at its tip. The drop‐shaped optical microfiber can exert microforce on biological specimens via the PDMS microsphere, and measure its deflection by monitoring the microfiber's transmittance. The PDMS microsphere does not only effectively isolate the waveguide evanescent field from the contact‐induced surface contamination, but also enables a stable contact between the sensor and the specimen, thereby facilitating quantitative analysis of biomechanical parameters via the Hertz contact model. Moreover, the incorporation of the PDMS microsphere greatly boosts the sensor's sensitivity. Theoretical modeling and experimental characterization confirm that this drop‐shaped optical microfiber is a linear elastic structure consistent with Hooke's law, featuring a tunable spring constant and operating force range achievable by modifying the fiber diameter and ring width. As proof‐of‐concept demonstrations, an optimized sensor with a resolution of 24 nN was employed to characterize Young's modulus of onion epidermal cells and live *Caenorhabditis elegans* (*C. elegans*), respectively. Capitalizing on its inherent advantages in sensitivity, miniaturization, flexibility, and mechanical robustness, this microforce sensor holds substantial potential for applications in biomechanics, cell mechanics, and materials science.

## 2 Results

### 2.1 Design and Fabrication of Drop‐Shaped Optical Microfiber Sensor

**FIGURE 1:**
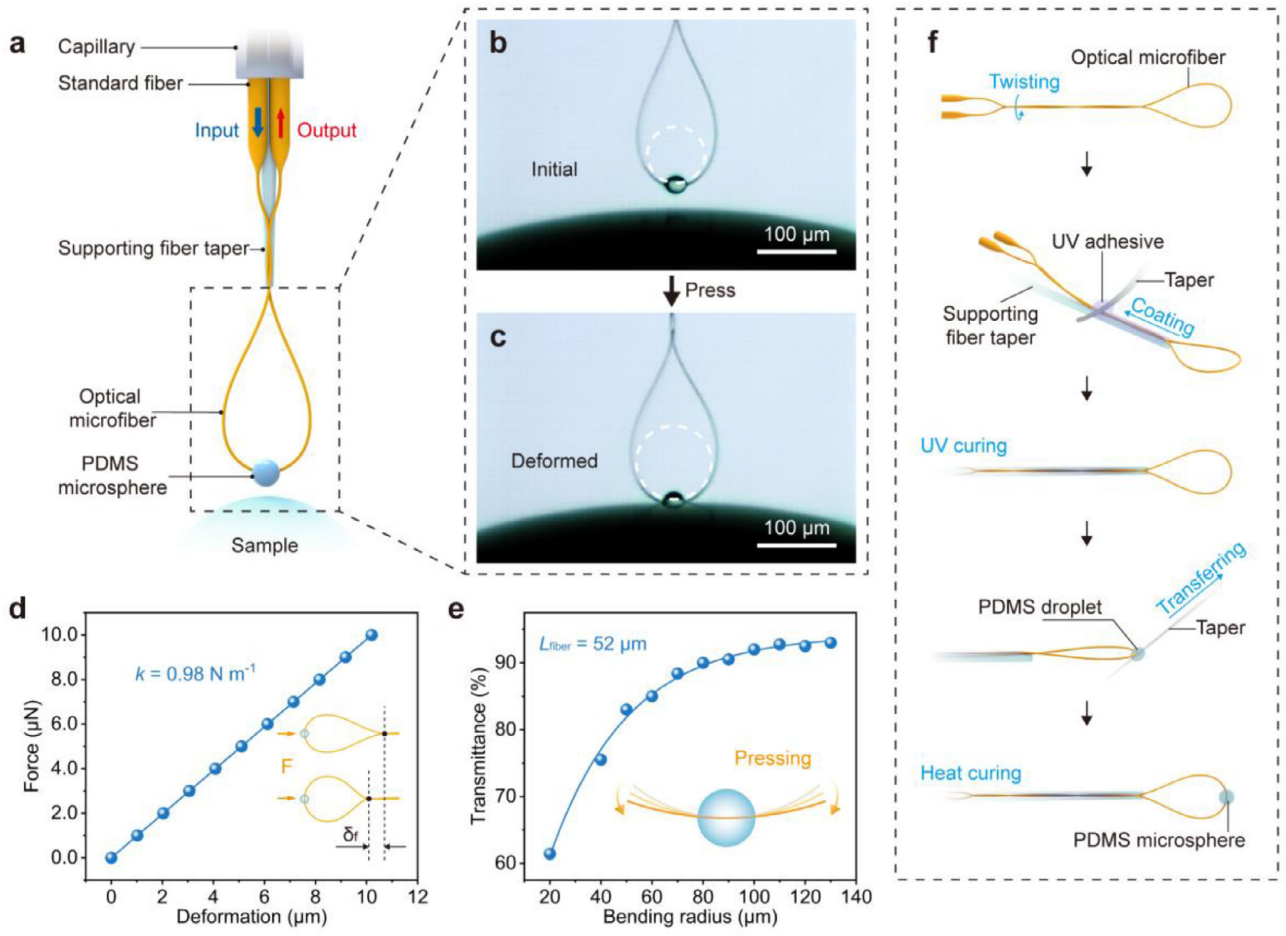
Design and fabrication of the drop‐shaped optical microfiber sensor. (a) Schematic illustration of a drop‐shaped optical microfiber sensor. (b,c) Microscope images of the drop‐shaped optical microfiber in its initial state (b) and deformed state (c). (d) Mechanical simulation of the relationship between deformation (*δ*_f_) and applied force (*F*) of a drop‐shaped optical microfiber (diameter: 2 µm; ring width: 125 µm). Inset: Definition of *δ*_f_. (e) Optical simulation of the relationship between bending radius and transmittance of a 2‐µm‐diameter optical microfiber. (f) Schematic illustration of the fabrication steps of the sensor.

Figure 1a schematically illustrates the architecture of the microforce sensor which comprises a drop‐shaped optical microfiber, a PDMS microsphere, a supporting fiber taper, and a silica capillary. The drop‐shaped optical microfiber is fabricated by a biconical tapered optical fiber, which consists of a taper waist (i.e., optical microfiber) with a uniform diameter, two transition regions with a gradually decreasing diameter, and two sections of standard optical fibers. A PDMS microsphere is located at the tip of the drop‐shaped optical microfiber. The taper transitions together with a section of taper waist are intimately contacted together, and mechanically stabilized by a supporting fiber taper with a diameter of ∼20 µm. A section of silica capillary (5 cm in length) is used to encapsulate the standard fibers and stabilize the supporting fiber taper, forming a drop‐shaped optical microfiber sensor (Figure S1). The standard optical fibers connect to a light source and a spectrometer/detector, respectively, serving as input/output ports for signal transmission. During the testing, the sensor presses orthogonally onto the sample surface via the PDMS microsphere. The displacement of the sensor pressing onto the sample is precisely controlled using a motorized displacement stage with a resolution of 0.1 µm (Figure S2). With the increasing of the sensor displacement, the bending radius of the drop‐shaped optical microfiber becomes larger (Figure 1b,c), which in turn results in a significant enhancement in transmittance of the optical microfiber.

To understand the relationship between the applied force and the optical transmittance, we performed mechanical and optical simulations. For a 125‐µm‐wide drop‐shaped optical microfiber composed of a 2‐µm‐diameter optical microfiber, the deformation (*δ*_f_) exhibits a linear response to the applied force within the range of 0–10 µN, resulting in a spring constant (*k*) of 0.98 N m^−1^ (Figure 1d). Thus, the drop‐shaped optical microfiber is a linear elastic structure that follows Hooke's law. Meanwhile, the optical simulation indicates that as the bending radius of the drop‐shaped optical microfiber increases, the transmittance of the optical microfiber increases exponentially (Figure 1e). Following calibration, *δ*_f_ can be derived by monitoring transmittance variations, and the applied force *F* can be calculated from *δ*_f_. Thus, we can deduce the sample's mechanical properties through transmittance monitoring.

Figure 1f schematically illustrates the fabrication process of the sensor. First, a biconical tapered optical fiber is folded at its midpoint, followed by twisting one of the standard optical fibers to bring the taper waist and taper transition of the two fiber ends into intimate contact. Second, the pre‐twisted optical microfiber is integrated with a supporting fiber taper using a thin layer of UV adhesive. After curing, the initial configuration of the drop‐shaped optical microfiber is secured. Third, the standard optical fibers at both ends of the drop‐shaped optical microfiber are encapsulated using a silica capillary with an inner diameter of 530 µm, and the supporting fiber taper is attached to the outer side of the capillary with UV adhesive. Finally, uncured PDMS droplets are deposited onto the tip of the drop‐shaped optical microfiber to form a PDMS microsphere (Figure S3), followed by curing at 80°C for 20 min. The size of PDMS microspheres can be tailored to meet the requirements of different samples (Figure S4).

### 2.2 Effects of the PDMS Microsphere

**FIGURE 2:**
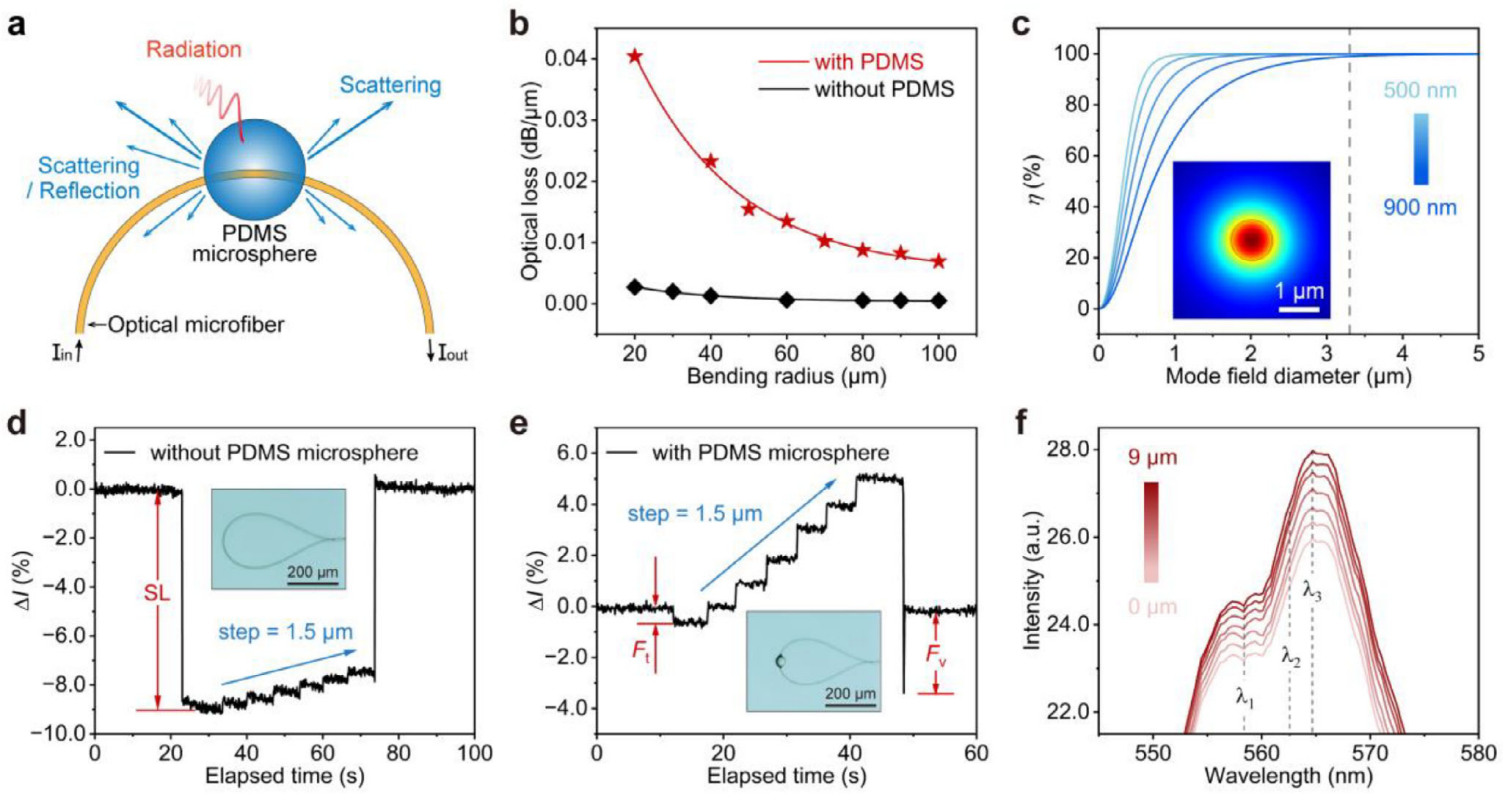
Effects of the PDMS microsphere on sensing performance. (a) Schematic illustration of the scattering, reflection, and radiation caused by the PDMS microsphere at the tip of the optical microfiber. (b) Comparison of bending loss in optical microfiber (diameter: 2 µm) with and without PDMS microsphere (diameter: 10 µm). (c) Power distribution of fundamental mode of a PDMS cladding optical microfiber (diameter: 1 µm) operated at wavelengths between 500 and 900 nm. Inset: Fundamental mode field of the 1‐µm‐diameter optical microfiber operated at wavelength 900 nm. (d,e) Output light intensity response to pressure without (d) and with (e) PDMS microsphere. Insets: Microscope images of the corresponding drop‐shaped optical microfibers. (f) Typical transmission spectra of a drop‐shaped optical microfiber integrated with a PDMS microsphere were recorded during stepwise pressing against a glass slide at intervals of 1.5 µm.

When light propagating through the optical microfiber encounters the PDMS microsphere, a portion of the light is scattered, reflected, or converted from guided modes to radiation modes (Figure 2a). It is worth noting that, although the additional loss introduced by the PDMS microsphere to a microfiber is nonnegligible (Figure S5), it will be significantly increased with bending curvature (i.e., 1/R_B_, where R_B_ is the bending radius) (Figure 2b), leading to a significant enhancement of the sensor's response to deformation. Moreover, the PDMS microsphere can effectively isolate the waveguide evanescent field of the optical microfiber from the surface of the sample, preventing the unwanted leakage and contamination caused by direct contact with samples. Once the fiber surface is contaminated during testing, ultrasonic cleaning can be employed to remove the contaminants. (Figure S6) Calculated optical power distribution of a PDMS‐clad optical microfiber (1 µm in diameter) operating across a wavelength range of 500–900 nm indicates that 99% of the waveguide power (900‐nm‐wavelength) is confined within a 3.3‐µm‐diameter mode field (Figure 2c). Therefore, a 20‐µm‐diameter PDMS microsphere can effectively isolate the evanescent field from the surface of the sample.

To verify the sensitivity enhancement by the PDMS microspheres, a drop‐shaped optical microfiber sensor without PDMS microsphere (inset of Figure 2d) was employed to press against the surface of a glass slide. When the optical microfiber contacted the glass slide, the output light intensity decreased significantly due to scattering loss (SL). As the sensor displacement increased, the bending loss of the optical microfiber decreased accordingly, resulting in a slight increase in output light intensity. When the optical microfiber was retracted from the glass slide, the output light intensity recovered to its initial value (Figure 2d). Subsequently, a 20‐µm‐diameter PDMS microsphere was deposited onto the optical microfiber sensor (inset of Figure 2e). When the sensor pressed the glass slide via the PDMS microsphere with the same step size of 1.5 µm (Movie S1), scattering loss upon the contact was much lower (approximately negligible) (Figure 2e), while the change in output intensity is significantly enlarged compared with that of the sensor without the PDMS microsphere. Note that the initial decrease in light intensity is attributed to the attractive surface force (*F*_t_) between the PDMS microsphere and the glass slide. This attractive force drove the microsphere to move toward the glass slide, resulting in a “jump‐to‐contact” event [48], which in turn induced reversibly stretching of the drop‐shaped optical microfiber and enhanced the bending loss (Figure 2e).

We investigated the effect of wavelength on the sensing performance. In the wavelength range of 530–570 nm, as the sensor displacement increased, the output light intensity increased obviously (Figure 2f). We defined the optical sensitivity of the sensor as *S*_O_ = Δ*I* / Δ*δ*_f_, where *I* (%) and *δ*_f_ (µm) represent the normalized output light intensity and sensor deformation, respectively. As shown in Figure S7, the sensor achieves the highest sensitive of 1.64% µm^−1^ at a wavelength of 564.7 nm, more than triple that of sensor without the PDMS microsphere (0.19% µm^−1^). Notably, when the PDMS microsphere became detached from the glass slide, a distinct signal spike was detected, which can be ascribed to the adhesive interaction force (*F*_v_) between the PDMS and the glass slide.

In addition, we investigated the effect of PDMS microsphere diameter on the sensing performance (Figure S8). The results show that the sensitivity of the sensor remains essentially unchanged when the diameter of the PDMS microsphere increases from 15 to 20 and 25 µm. Besides PDMS, UV curable fluoropolymer (EFIRON PC‐373) can also be used to fabricate microspheres at the apex of the microfiber without exerting a significant influence on sensing performance (Figure S9). However, owing to its different surface properties compared with PDMS, the indentation curves obtained by this sensor exhibit certain differences from that of the PDMS microsphere sensor (Figure S10).

### 2.3 Sensing Performance

**FIGURE 3:**
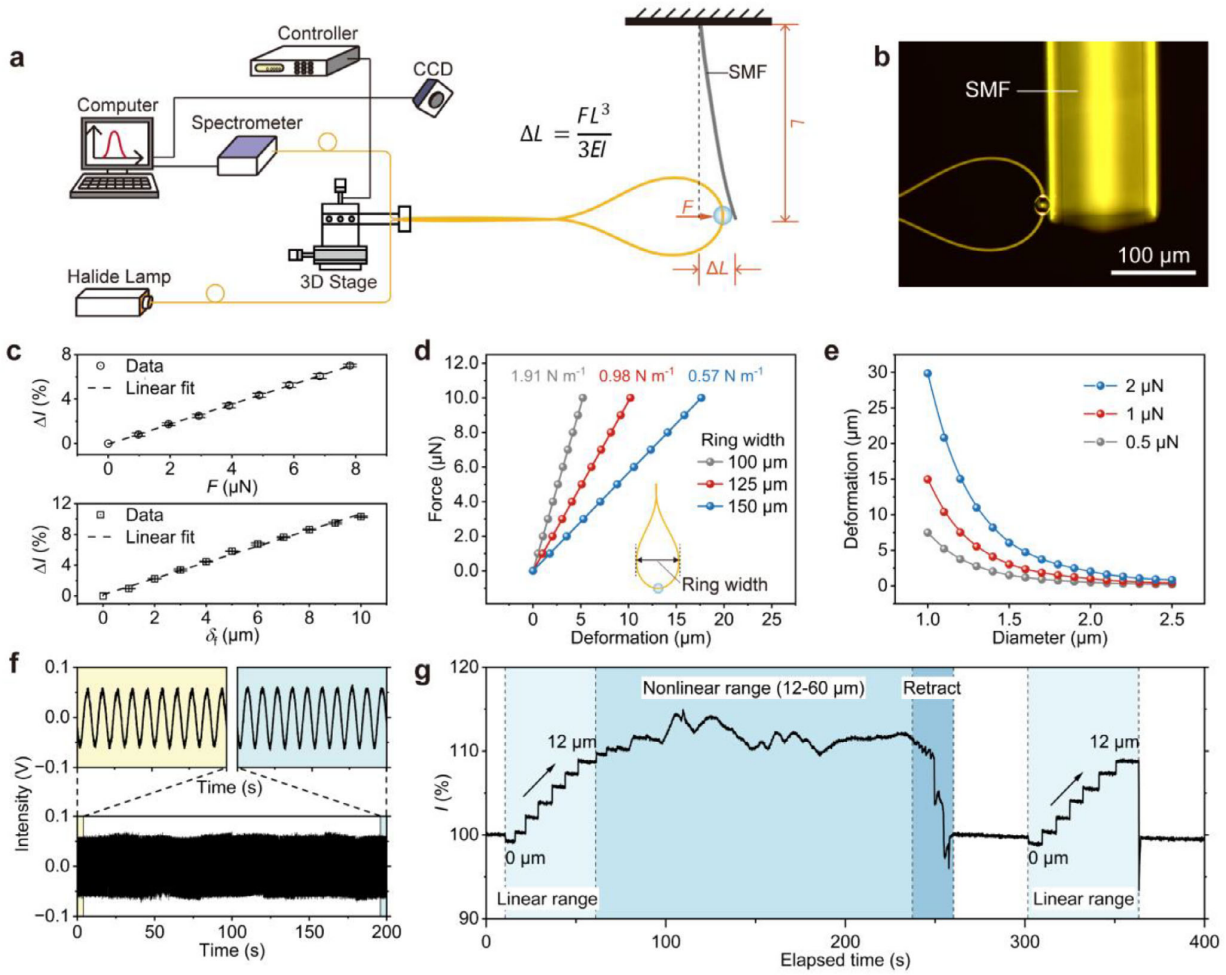
Characterization of the drop‐shaped optical microfiber sensor. (a) Schematic of the experimental setup for calibrating the sensor. (b) Microscope image of the drop‐shaped optical microfiber pressing at the free end of a standard SMF cantilever. (c) Plots of output light intensity change (Δ*I*) versus applied force (*F*) (Top) and sensor deformation (*δ*_f_) (Bottom). (d) Mechanical simulation showing the effect of ring width of the drop‐shaped optical microfiber (diameter: 2 µm) on its spring constant. The inset depicts the definition of the microfiber's ring width. (e) Mechanical simulation showing the effect of the optical microfiber diameter on the sensor deformation of a drop‐shaped optical microfiber (width: 125 µm). (f) The response of the sensor under sinusoidal load over more than 100 000 cycles. (g) Plot of the sensor's linear and nonlinear responses over a pressing distance of 0–60 µm.

In this study, the spring constant of the drop‐shaped optical microfiber was calibrated using a vertically suspended single‐mode fiber (SMF) cantilever. During the calibration process, the sensor's displacement was controlled by a motorized 3‐axis stage, the real‐time transmission spectrum was recorded by a fiber spectrometer, and the deflection of the SMF was captured by a CCD camera (Figure 3a). When the sensor presses orthogonally against the free end of the SMF (Figure 3b; Movie S2), both the sensor and the SMF undergo elastic deflection. The deflection of the SMF (Δ*L*) was obtained from microscopic imaging and the applied force (*F*) was calculated according to the beam deflection formula (Equation (1)):

(1) $$\Delta L=\frac{FL^{3}}{3EI}$$

where *L*, *E*, and *I* represent the length, Young's modulus, and sectional moment of inertia of the SMF, respectively. Here, *L* is 40 mm, *E* is 73 GPa, and *I* can be calculated using the circular cross‐section moment of inertia formula (Equation (2)):

(2) $$I=\frac{\pi d^{4}}{64}$$

where the diameter of the SMF (*d*) is 125 µm. For an SMF with a length of 40 mm, a 1‐µm‐deflection corresponds to an applied force of 0.04 µN. As shown in Figure 3c, the change of output light intensity (Δ*I*) exhibits a linear relationship with the applied force (Equation (3)):

(3) ΔI=0.90F

Meanwhile, when the sensor pressing against a glass slide with a step of 1 µm (Figure 3c), Δ*I* shows a good linear relationship with the sensor deformation (*δ*_f_) (Equation (4)):

(4) $$\Delta I=1.05\delta_{f}$$

Consequently, we obtain the force‐deformation relation for the sensor (Equation (5)):

(5) $$F=1.17\delta_{f}$$

This yields a spring constant of 1.17 N m^−1^, which is comparable to that of AFM cantilevers [11], making it a promising candidate for microforce sensing. Typically, a drop‐shaped optical microfiber with a larger ring width and thinner diameter exhibits a lower spring constant (Figure 3d,e). For the 120‐µm‐width drop‐shaped optical microfiber (diameter: 2 µm), its mechanical sensitivity (*S*) was calculated as *S* = Δ*I* / Δ*F* = 1.87% µN^−1^ (Figure S11). Given the sensor's static noise of 0.045% (Figure S12), its resolution was calculated to be 24 nN. To test the sensor's reversibility, it was subjected to a sinusoidal load at 0.5 kHz. After over 100 000 cycles, the output intensity showed excellent repeatability, with a relative standard deviation (RSD) of 4.67% (Figure 3f). Moreover, the sensor can respond rapidly to dynamic stimuli with a frequency up to 1.5 kHz (Figure S13), which is crucial for the detection of dynamic biological processes in living organisms.

To investigate the operating range of the sensor, we gradually pressed the sensor on a glass slide over a displacement range from 0 to 60 µm, and recorded the corresponding changes in the sensor's output signal (Figure 3g). The sensor exhibited a linear response in the first range of 0–12 µm, whereas became non‐linear in the second range of 12–60 µm, due to the irregular bending. After the sensor was retracted and detached from the glass slide, its output signal returned to the initial value. When the sensor was repressed onto the glass slide, it repeated a same linear response in the first range of 0–12 µm (Movie S3), indicating that the sensor is less prone to damage even under deformation beyond its typical operating range, which is favorable for measuring biological samples with relatively high surface viscosity.

### 2.4 Measurement of Young's Modulus of Onion Cells

**FIGURE 4:**
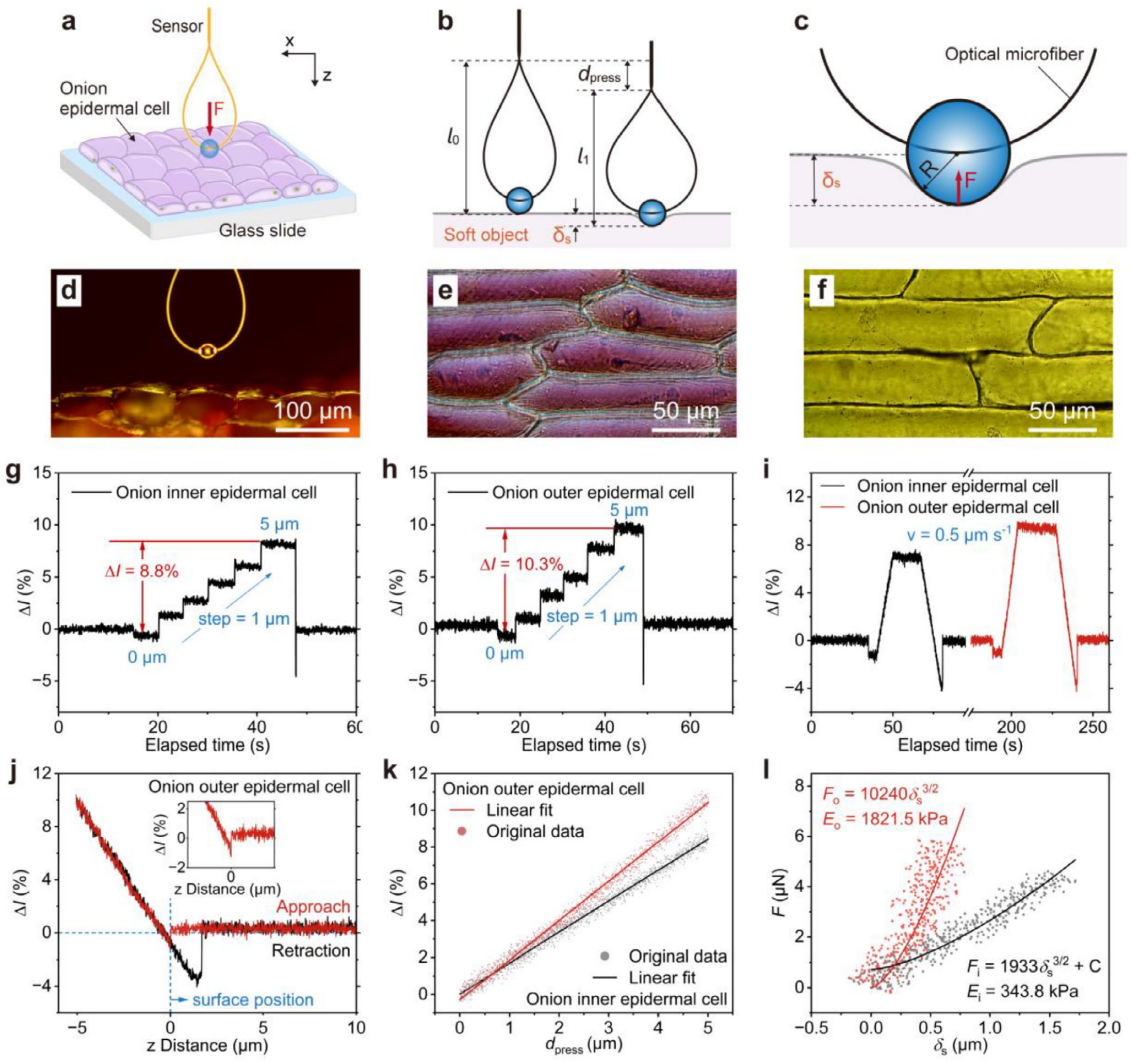
Measuring Young's modulus of onion epidermal cells. (a) Schematic of the sensor pressing on onion epidermal cells. (b) Schematic showing the deformation of the sensor and the sample. (c) Schematic illustration of Hertz contact model. (d) Microscope image of the sensor and onion epidermal cells. (e,f) Microscope images of onion outer (e) and inner (f) epidermal cells. (g,h) Responses of the sensor to onion inner (g) and outer (h) epidermal cells, respectively. (i) Light intensity changes during the sensor's approach to, pressing on, and retraction from the onion inner (black) and outer (red) epidermal cell surfaces at a constant speed. (j) Light intensity change (extracted from (i)) versus the sensor's vertical distance during approach to and retraction from an onion outer epidermal cell. Inset: Magnified view of the negative peak observed during the approach phase. (k) Light intensity changes versus *d*_press_ for interactions with onion inner and outer epidermal cells, respectively. (l) *F*‐*δ*_s_ curves for onion outer (red) and inner (black) epidermal cells. Solid lines represent the corresponding fits with the Hertz contact model.

To validate the capability of the sensor for measuring Young's modulus of individual cells, we selected the onion epidermal cell as the test sample (Figure 4a). Using a microscopic imaging system and a precise motorized displacement stage, the sensor was gradually pressed onto a single onion epidermal cell, while the change in the output light intensity of the microfiber was monitored. During the pressing process, both the sensor and the cell underwent varying degrees of deformation. As shown in Figure 4b, *l*_0_ +   *δ*_s_ =  *l*_1_  +  *d*_press_, where *l*_0_ and *l*_1_ denote the initial and deformed lengths of the drop‐shaped optical microfiber, respectively; *δ*_s_ represents the deformation of the sample; and *d*_press_ refers to sensor displacement during the indentation phase. Given that the deformation of the drop‐shaped optical microfiber is *δ*_f_ =  *l*_0_ −  *l*_1_, the deformation of the sample can be calculated as *δ*_s_ =  *d*_press_ −  *δ*_f_. The applied force (*F*) and *δ*_f_ can be derived from Δ*I* using the calibrated Δ*I*‐*F*‐*δ*_f_ functional relationship. Additionally, the PDMS microsphere acts as the direct contact medium. Since the Young's modulus of PDMS is significantly higher than that of the measured sample (e.g., onion epidermal cells and *C. elegans*), and the equivalent stiffness of the PDMS microsphere is considerably greater than that of the microfiber ring, the deformation of the PDMS microsphere can be neglected when the sensor is pressed against soft biological samples. Its geometric structure approximates that of a standard sphere, thereby facilitating the quantitative measurement (Figure 4c). Finally, we can calculate the Young's modulus *E* using the Hertz contact model [49, 50] (Equation (6)) for spherical probes.

(6) $$F=\frac{4\sqrt{R}}{3}\cdot \frac{E_{s}}{\left(1-\nu^{2}\right)}\cdot {\delta_{s}}^{3/2}$$

where *R*, *E*_s_ and *ν* represent radius of the spherical probe, Young's modulus of the sample, and Poisson ratio, respectively.

Experimentally, we employed a 110‐µm‐wide sensor with a 20‐µm‐diameter PDMS microsphere (Figure 4d) to measure the Young's modulus of onion epidermal cells, typically, ∼40 µm in width and ∼100 µm in length (Figure 4e,f). During the indentation process, when the sensor displacement was set to 5 µm with an increment of 1 µm, the changes of light intensity in the outer epidermal cells were larger than those in the inner epidermal cells (Figure 4g,h), indicating that the outer epidermal cells of onion exhibit higher Young's modulus. Calculations reveal that a 1‐µm sensor displacement corresponds to an applied force of approximately 1 µN. Over a total displacement of 5 µm, the maximum deformation in the outer and inner epidermal cells were approximately 0.8 and 1.4 µm, respectively.

To further investigate the mechanical response of onion epidermal cells, we performed an approach‐retract test. The sensor was set at a constant speed (0.5 µm s^−1^) to approach to and retract from the cell, and the real time output light signal was recorded (Figure 4i). Figure 4j illustrates the relationship between the resulting change in light intensity (Δ*I*) and the sensor's vertical distance (z distance), with the cell surface position defined as zero. The red curve in Figure 4j represents the sensor approach phase, where small negative peaks indicate snap‐on contact events between the PDMS microsphere and the cell surface. Following contact initiation, light intensity increased linearly with indentation depth.

During sensor retraction (black curve), intensity decreased at the same slope as the loading curve, with both trajectories nearly overlapping. This high reversibility indicates predominantly elastic deformation of the cells, which rapidly recovered upon load removal. Notably, the light intensity continued to decrease into negative values at the end of the retraction curve. This is attributed to the adhesive force (∼1.94 µN) between the PDMS microsphere and the cell, which stretched the drop‐shaped optical microfiber, thereby enhancing the bending loss until complete separation, after which the drop‐shaped optical microfiber recovered its initial value. A comparative analysis reveals that outer epidermal cells exhibit a higher rate of light intensity change (Figure 4k). For quantitatively comparison of cellular stiffness, the Δ*I*‐*d*_press_ data were converted into the relationship between the applied force (*F*) and cellular deformation (*δ*_s_) using the pre‐calibrated Δ*I*‐*F*‐*δ*_f_ function. The resulting *F*‐*δ*_s_ curve (Figure 4l) was then fitted with the Hertz contact model to calculate the Young's modulus of the cells. Statistical analysis of multiple groups of inner and outer onion epidermal cells yielded Young's moduli of 520 ± 170 kPa (inner epidermal cells) and 1800 ± 941 kPa (outer epidermal cells), respectively (Figure S14, Table S1), showing good agreement with previously reported values ranging from 200 to 4900 kPa [51].

### 2.5 Measurement of Young's Modulus of C. Elegans

**FIGURE 5:**
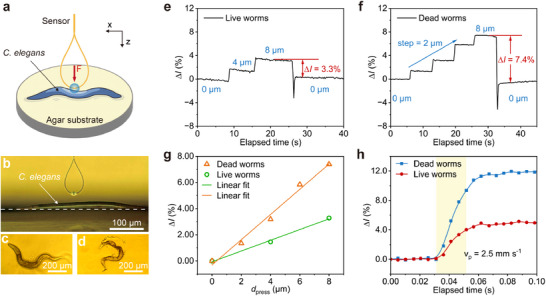
Measuring Young's modulus of *C. elegans* using a drop‐shaped optical microfiber sensor. (a) Schematic diagram of the sensor pressing on a worm. (b) Microscope image of the sensor and a worm. (c,d) Microscope images of a live worm (c) and a dead worm (d). (e,f) Light intensity change (Δ*I*, %) of the sensor as a function of elapsed time (s) during indentation tests with live worms (e) and dead worms (f). (g) Light intensity change versus *d*_press_ for indentation with live and dead worms, respectively. (h) Light intensity change (Δ*I*, %) of the sensor as a function of elapsed time (s) during approach‐indentation tests with live worms and dead worms. The yellow shaded region indicates the linear response range.

To explore the potential of this sensor for in vivo Young's modulus measurements, we selected the *unc‐73* [52] strain of *C. elegans* as a model organism (Figure 5a,b). To minimize the influence of worms’ movement on testing, we employed ultraviolet illumination to reduce worms’ locomotor activity. We tested both the live (Figure 5c) and dead (Figure 5d) worms, and compared their Young's moduli. Optical microscopic images clearly showed that the live worms appeared plump and elastic, whereas the dead ones were rigid due to dehydration. Similar to the tests performed on the onion epidermal cells, we conducted indentation tests on the worms (Figure 5e–g). When the sensor indented the live worm with a displacement of 8 µm, the worm underwent approximately 6.3 µm deformation while the drop‐shaped optical microfiber experienced only 1.7 µm deformation, resulting in a light intensity change of merely 3.3%. During indentation of the dead worm, the light intensity change reached 7.4% at a sensor displacement of 8 µm. This indicates a larger deformation of the drop‐shaped optical microfiber, and correspondingly larger Young's modulus in the dead worm. Calculations yielded Young's modulus values of 31.1 ± 7.9 kPa for live worms, which are matched well with the reported results of ∼30 kPa [53, 54], and 215.1 ± 109.7 kPa for dead worms (Figure S14, Table S1). Furthermore, we performed an approach‐indentation test. To mitigate the peristalsis of live worms on the measurement, the sensor was set a speed of 2.5 mm s^−1^ to approach and indent the worm (Figure 5h). As expected, the light intensity change rate associated with live worms was lower than that of dead ones, indicating that live worms are softer than dead individuals.

In addition, we investigated the influence of non‐perpendicular contact between the sensor and the sample on the measurement results (Figure S15). To minimize the undesirable influence of sensor tilting, prependicular contact between the sample and the sensor should be maintained as much as possible during measurements by virtue of the microscopic imaging system. Furthermore, repeated measurements at adjacent positions can mitigate errors induced by surface curvature variations.

## 3 Conclusion

In summary, we have developed a drop‐shaped optical microfiber microforce sensor for biomechanical sensing. Leveraging its unique mechanical properties, the drop‐shaped optical microfiber sensor features a small footprint (ring width < 120 µm), low spring constant (0.9 N m^−^^1^), and a large deformation range (0–60 µm), rendering it exceptional flexibility and robustness for characterizing biological specimens. On the other hand, with the presence of the PDMS microsphere, bending‐radius‐dependent output light intensity enables nanonewton‐level force sensing through monitoring variations in output light intensity, making the drop‐shaped optical microfiber a self‐sensing structure. In addition, the PDMS microsphere not only effectively isolates the evanescent field from the environment, preventing microfiber surface contamination, but also promotes a more compliant sensor‐sample contact, which aligns better with the Hertz contact model and thereby improves the accuracy of measured mechanical properties. Theoretical analyses and experimental results confirmed an excellent linear response within the 0–10 µN force range, yielding a sensitivity of 1.87% µN^−^^1^ and a force resolution of 24 nN. As proof‐of‐concept demonstrations, Young's modulus measurements in both single cells and *C. elegans* were realized.

Compared with AFM, optical tweezers and magnetic tweezers, our proposed sensor features a simple structure and flexible operation, and effectively eliminates the damage to biological samples caused by AFM probes and high‐intensity laser irradiation. In contrast to fiber‐tip FP cavity sensors, our sensor possesses a facile fabrication process and excellent robustness, without the requirement of sophisticated 3D printing techniques. It achieves an ultra‐large deformation range, and can fully recover to its initial state even after undergoing deformations several times beyond its operational range. Such performance is particularly crucial for the mechanical characterization of highly viscous biological samples. Our sensor is applicable not only to measurements on samples with dry surfaces, but also to samples covered with water or normal saline (Figure S16), which is crucial for expanding its applications in biology, medicine, and materials science. Furthermore, the sensor can be repeatedly reused for long‐term measurements via ultrasonic cleaning once the fiber surface is contaminated. In addition to quantifying the Young's modulus of biological specimens, our sensor is also able to measure adhesion force, micro‐strain, and micro‐friction at microscale and mesoscale interfaces. We envision this sensor, built with optical microfiber and a PDMS microsphere as construction units, providing a promising tool for biomechanics, biomaterials, and micro‐nano materials.

## 4 Experimental Section

### 4.1 Preparation of Biconical Optical Microfiber

Biconical optical microfibers were fabricated from standard optical fibers (62.5/125 µm, Corning) via a flame‐heated mechanical stretching method. A section of the optical fiber coating was stripped, followed by fixing the fiber on the fiber taper instrument. When the fiber was heated to its softening temperature, two computer‐controlled translation stages was used to stretch it at a velocity of 0.1 mm s^−1^, gradually reducing the diameter from 125 µm to the target diameter. To precisely control its diameter, the real‐time transmission of the optical fiber was monitored during the stretching process.

### 4.2 Deposition of PDMS Microspheres at the Tip of Drop‐Shaped Optical Microfiber

PDMS pre‐polymer and curing agent were mixed at a 5:1 ratio. A fiber taper was dipped into the uncured PDMS mixture to attach quite a few liquid droplets (Figure S17). Typically, the volume of the droplets varies from 20 to 200 fL. Subsequently, individual PDMS droplet was sequentially deposited onto the drop‐shaped optical microfiber from the fiber taper via a micromanipulation platform, followed by curing at 80°C for 20 min. The process of transferring PDMS droplets was carried out under an optical microscope (Nikon Eclipse ME600P, Tokyo, Japan). The diameter of the PDMS microspheres was measured, and the transfer of PDMS droplets was stopped once the target diameter was achieved. Theoretically, the diameter control precision of this method can be better than 0.5 µm.

### 4.3 Experimental Setup

A halide light (SLS201L/M, THORLABS, USA) was employed as light source; an optical fiber spectrometer (LEDPRO‐50, Ocean Optics, USA) was used to capture the output intensity of the sensor; the displacement of the sensor was controlled by a 3D translation stage (Model ESP301, Newport, USA); the microscope images were obtained by a microscope equipped with an objective lens (M plan Apo 20x, Mitutoyo, Japan) and a CCD camera (MER‐1070‐14U3C‐L, Da Heng, China). To characterize the sensor's dynamic response, a vibrating horn was employed for vibration transmission; a signal generator (UTG2122B, UNI‐T, China) controlled vibrating horn was used to generate high‐frequency sinusoidal stimuli to the sensor; a laser diode (CLD1010LP, THORLABS, Germany) and an oscilloscope (MDO34, Tektronix, China) were used as light source and detector, respectively.

### 4.4 Strains and Culture of C. Elegans

All strains were maintained at 20°C on NGM (nematode growth medium) plates seeded with Escherichia coli OP50, using standard conditions as previously described [55, 56]. Nematode Growth Medium (NGM) components include NaCl (3.0 g L^−1^), agar (20.0 g L^−1^), peptone (2.5 g L^−1^), 1 mm MgSO_4_, 1 mm CaCl_2_, 5 µg mL^−1^ cholesterol, and 1 m K_2_HPO_4_‐KH_2_PO_4_ buffer (25 mL L^−1^). Strains were obtained by Xiaomin Yue Lab. The wild‐type strain is Bristol N2 strain from Caenorhabditis Genetics Center (CGC), and the mutant strain is *unc‐73(e936)I*.

## Author Contributions

L.Z., L.T. and X.Y. supervised the project and revised the manuscript. Y.X. and X.T. designed the sensor structure. Y.X. carried out the experiments, analyzed the results, wrote the manuscript, and prepared the figures. Y.X., X.T., and X.Z. conducted the theoretical analysis and simulations. All authors discussed the results and assisted in the preparation of the manuscript.

## Conflicts of Interest

The authors declare no conflicts of interest.

## Supporting information




**Supporting File 1**: advs75673‐sup‐0001‐SuppMat.docx.

**Supporting File 2**: advs75673‐sup‐0002‐MovieS1‐S3.zip.

## Data Availability

The data that support the findings of this study are available from the corresponding author upon reasonable request.

## References

[1] D. Wirtz , K. Konstantopoulos , and P. C. Searson , “The Physics of Cancer: The Role of Physical Interactions and Mechanical Forces in Metastasis,” Nature Reviews Cancer 11 (2011): 512–522, 10.1038/nrc3080. 21701513 PMC3262453

[2] S. Kumar and V. Weaver , “Mechanics, Malignancy, and Metastasis: The Force Journey of a Tumor Cell,” Cancer and Metastasis Reviews 28 (2009): 113–127, 10.1007/s10555-008-9173-4. 19153673 PMC2658728

[3] A. Magazzù and C. Marcuello , “Investigation of Soft Matter Nanomechanics by Atomic Force Microscopy and Optical Tweezers: A Comprehensive Review,” Nanomaterials 13 (2023): 963, 10.3390/nano13060963. 36985857 PMC10053849

[4] R. Garcia , “Nanomechanical Mapping of Soft Materials With the Atomic Force Microscope: Methods, Theory and Applications,” Chemical Society Reviews 49 (2020): 5850–5884, 10.1039/D0CS00318B. 32662499

[5] A. Lostao , K. Lim , M. C. Pallarés , A. Ptak , and C. Marcuello , “Recent Advances in Sensing the Inter‐Biomolecular Interactions at the Nanoscale—A Comprehensive Review of AFM‐Based Force Spectroscopy,” International Journal of Biological Macromolecules 238 (2023): 124089, 10.1016/j.ijbiomac.2023.124089. 36948336

[6] T. Li , S. Yu , B. Sun , et al., “Bioinspired Claw‐Engaged and Biolubricated Swimming Microrobots Creating Active Retention in Blood Vessels,” Science Advances 9 (2023): adg4501, 10.1126/sciadv.adg4501. PMC10162671 37146139

[7] H. Xie , M. Sun , X. Fan , et al., “Reconfigurable Magnetic Microrobot Swarm: Multimode Transformation, Locomotion, and Manipulation,” Science Robotics 4 (2019): aav8006, 10.1126/scirobotics.aav8006. 33137748

[8] Z. Mai , Y. Lin , P. Lin , X. Zhao , and L. Cui , “Modulating Extracellular Matrix Stiffness: A Strategic Approach to Boost Cancer Immunotherapy,” Cell Death & Disease 15 (2024): 307, 10.1038/s41419-024-06697-4. 38693104 PMC11063215

[9] M. A. Wozniak and C. S. Chen , “Mechanotransduction in Development: A Growing Role for Contractility,” Nature Reviews Molecular Cell Biology 10 (2009): 34–43, 10.1038/nrm2592. 19197330 PMC2952188

[10] D. E. Discher , P. Janmey , and Y. L. Wang , “Tissue Cells Feel and Respond to the Stiffness of Their Substrate,” Science 310 (2005): 1139–1143, 10.1126/science.1116995. 16293750

[11] D. H. Cho , S. Aguayo , and A. X. Cartagena‐Rivera , “Atomic Force Microscopy‐Mediated Mechanobiological Profiling of Complex Human Tissues,” Biomaterials 303 (2023): 122389, 10.1016/j.biomaterials.2023.122389. 37988897 PMC10842832

[12] W. Liang , H. Shi , X. Yang , et al., “Recent Advances in AFM‐Based Biological Characterization and Applications at Multiple Levels,” Soft Matter 16 (2020): 8962–8984, 10.1039/D0SM01106A. 32996549

[13] M. Krieg , G. Fläschner , D. Alsteens , et al., “Atomic Force Microscopy‐Based Mechanobiology,” Nature Reviews Physics 1 (2019): 41–57, 10.1038/s42254-018-0001-7.

[14] Y. Abidine , A. Constantinescu , V. M. Laurent , et al., “Mechanosensitivity of Cancer Cells in Contact With Soft Substrates Using AFM,” Biophysical Journal 114 (2018): 1165–1175, 10.1016/j.bpj.2018.01.005. 29539402 PMC5883622

[15] M. H. Jo , P. Meneses , O. Yang , C. C. Carcamo , S. Pangeni , and T. Ha , “Determination of Single‐Molecule Loading Rate During Mechanotransduction in Cell Adhesion,” Science 383 (2024): 1374–1379, 10.1126/science.adk6921. 38513010 PMC10977658

[16] C. J. Bustamante , Y. R. Chemla , S. X. Liu , and M. D. Wang , “Optical Tweezers in Single‐Molecule Biophysics,” Nature Reviews Methods Primers 1 (2021): 25, 10.1038/s43586-021-00021-6. PMC8629167 34849486

[17] H. Zhang and K. K. Liu , “Optical Tweezers for Single Cells,” Journal of the Royal Society Interface 5 (2008): 671–690, 10.1098/rsif.2008.0052. 18381254 PMC2408388

[18] M. Zhu , K. W. Zhang , H. Tao , S. Hopyan , and Y. Sun , “Magnetic Micromanipulation for In Vivo Measurement of Stiffness Heterogeneity and Anisotropy in the Mouse Mandibular Arch,” Research 2020 (2020): 7914074, 10.34133/2020/7914074. 32666052 PMC7327709

[19] M. Zhu , H. Tao , M. Samani , et al., “Spatial Mapping of Tissue Properties In Vivo Reveals a 3D Stiffness Gradient in the Mouse Limb Bud,” Proceedings of the National Academy of Sciences 117 (2020): 4781–4791, 10.1073/pnas.1912656117. PMC7060671 32071242

[20] F. Serwane , A. Mongera , P. Rowghanian , et al., “In Vivo Quantification of Spatially Varying Mechanical Properties in Developing Tissues,” Nature Methods 14 (2017): 181–186, 10.1038/nmeth.4101. 27918540 PMC5524219

[21] F. Yuan , H. Qi , B. Song , et al., “Tailorable Biosensors for Real‐Time Monitoring of Stress Distribution in Soft Biomaterials and Living Tissues,” Nature Communications 16 (2025): 1081, 10.1038/s41467-025-56422-8. PMC11772616 39870637

[22] A. Meijerink , “Light Turns Tiny Crystals Into Force Sensors,” Nature 637 (2025): 35–36, 10.1038/d41586-024-04103-9. 39743597

[23] A. Meijerink and F. T. Rabouw , “Giant Photon Avalanches Observed in Nanoparticles,” Nature 589 (2021): 204–205, 10.1038/d41586-020-03659-6. 33442036

[24] R. Xie , F. Han , Q. Yu , et al., “A Movable Long‐Term Implantable Soft Microfibre for Dynamic Bioelectronics,” Nature 645 (2025): 648–655, 10.1038/s41586-025-09344-w. 40962980

[25] Y. Cui , W. H. Leong , G. L. Zhu , R. B. Liu , and Q. Li , “Nanodiamond‐Based Spatial–Temporal Deformation Sensing for Cell Mechanics,” ACS Nano 19 (2025): 13740–13751, 10.1021/acsnano.4c15003. 40175883 PMC12004926

[26] Q. Zheng , M. Peng , Z. Liu , et al., “Dynamic Real‐Time Imaging of Living Cell Traction Force by Piezo‐Phototronic Light Nano‐Antenna Array,” Science Advances 7 (2021): abe7738, 10.1126/sciadv.abe7738. PMC8153726 34039600

[27] D. Iannuzzi , S. Deladi , J. W. Berenschot , S. de Man , K. Heeck , and M. C. Elwenspoek , “Fiber‐Top Atomic Force Microscope,” Review of Scientific Instruments 77 (2006): 106105, 10.1063/1.2358710.

[28] M. Zou , C. Liao , S. Liu , et al., “Fiber‐Tip Polymer Clamped‐Beam Probe for High‐Sensitivity Nanoforce Measurements,” Light: Science & Applications 10 (2021): 171, 10.1038/s41377-021-00611-9. PMC8397746 34453031

[29] M. Zou , C. Liao , Y. Chen , et al., “3D Printed Fiber‐Optic Nanomechanical Bioprobe,” International Journal of Extreme Manufacturing 5 (2023): 015005, 10.1088/2631-7990/acb741.

[30] X. Shang , N. Wang , S. Cao , et al., “Fiber‐Integrated Force Sensor using 3D Printed Spring‐Composed Fabry‐Perot Cavities With a High Precision Down to Tens of Piconewton,” Advanced Materials 36 (2024): 2305121, 10.1002/adma.202305121. 37985176

[31] L. Zhang , Y. Tang , and L. M. Tong , “Micro‐/Nanofiber Optics: Merging Photonics and Material Science on Nanoscale for Advanced Sensing Technology,” iscience 23 (2020): 100810. 31931430 10.1016/j.isci.2019.100810 PMC6957875

[32] X. Guo , Y. Ying , and L. Tong , “Photonic Nanowires: From Subwavelength Waveguides to Optical Sensors,” Accounts of Chemical Research 47 (2014): 656–666, 10.1021/ar400232h. 24377258

[33] J. Y. Lou , L. M. Tong , and Z. Z. Ye , “Modeling of Silica Nanowires for Optical Sensing,” Optics Express 13 (2005): 2135–2140, 10.1364/OPEX.13.002135. 19495101

[34] L. Tong , R. R. Gattass , J. B. Ashcom , et al., “Subwavelength‐Diameter Silica Wires for Low‐Loss Optical Wave Guiding,” Nature 426 (2003): 816–819, 10.1038/nature02193. 14685232

[35] S. Ma , X. Wang , P. Li , et al., “Optical Micro/Nano Fibers Enabled Smart Textiles for Human–Machine Interface,” Advanced Fiber Materials 4 (2022): 1108–1117.

[36] L. Zhang , J. Pan , Z. Zhang , et al., “Ultrasensitive Skin‐Like Wearable Optical Sensors Based on Glass Micro/Nanofibers,” Opto‐Electronic Advances 3 (2020): 190022, 10.29026/oea.2020.190022.

[37] Y. Tang , H. Liu , J. Pan , et al., “Optical Micro/Nanofiber‐Enabled Compact Tactile Sensor for Hardness Discrimination,” ACS Applied Materials & Interfaces 13 (2021): 4560–4566, 10.1021/acsami.0c20392. 33435667

[38] J. Zhu , Y. Tong , Z. Wang , et al., “Gold Flake‐Enabled Miniature Capacitive Picobalances,” Small Methods 9 (2024): 2401640, 10.1002/smtd.202401640. 39659074

[39] D. W. Cai , T. Tong , Z. Zhang , J. Pan , L. Zhang , and L. M. Tong , “Functional Film Coated Optical Micro/Nanofibers for High‐Performance Gas Sensing,” IEEE Sensors Journal 19 (2019): 9229–9234, 10.1109/JSEN.2019.2924596.

[40] J. H. Li , J. H. Chen , and F. Xu , “Sensitive and Wearable Optical Microfiber Sensor for Human Health Monitoring,” Advanced Materials Technologies 3 (2018): 1800296, 10.1002/admt.201800296.

[41] H. Li , Y. Huang , G. Hou , et al., “Single‐Molecule Detection of Biomarker and Localized Cellular Photothermal Therapy Using an Optical Microfiber With Nanointerface,” Science Advances 5 (2019): aax4659, 10.1126/sciadv.aax4659. PMC6991926 32064314

[42] L. Yang , Y. Li , F. Fang , et al., “Highly Sensitive and Miniature Microfiber‐Based Ultrasound Sensor for Photoacoustic Tomography,” Opto‐Electronic Advances 5 (2022): 200076, 10.29026/oea.2022.200076.

[43] Y. Z. Liang , H. J. Sun , L. H. Cheng , L. Jin , and B. O. Guan , “High Spatiotemporal Resolution Optoacoustic Sensing With Photothermally Induced Acoustic Vibrations in Optical Fibres,” Nature Communications 12 (2021): 4139, 10.1038/s41467-021-24398-w. PMC8260642 34230467

[44] J. Yu , L. Chen , H. Dong , et al., “Sensing and Exploiting Static Femto‐Newton Optical Forces by a Nanofiber With White‐Light Interferometry,” ACS Photonics 5 (2018): 3205–3213, 10.1021/acsphotonics.8b00450.

[45] W. Yu , J. Zhu , Y. Xu , et al., “Optical Nanofiber‐Enabled Self‐Detection Picobalance,” ACS Photonics 11 (2024): 2316–2323, 10.1021/acsphotonics.4c00216.

[46] L. Chen , B. Liu , C. Markwell , et al., “A Nanonewton Force Sensor Using a U‐Shape Tapered Microfiber Interferometer,” Science Advances 10 (2024): adk8357, 10.1126/sciadv.adk8357. PMC11135392 38809971

[47] Y. Chen , S. C. Yan , X. Zheng , F. Xu , and Y. Q. Lu , “A Miniature Reflective Micro‐Force Sensor Based on a Microfiber Coupler,” Optics Express 22 (2014): 2443–2450, 10.1364/OE.22.002443. 24663535

[48] B. Cappella and G. Dietler , “Force‐Distance Curves by Atomic Force Microscopy,” Surface Science Reports 34 (1999): 5–104, 10.1016/S0167-5729(99)00003-5.

[49] H. Hertz , “Ueber die Berührung Fester Elastischer Körper,” crll 1882 (1882): 156–171, 10.1515/crll.1882.92.156.

[50] P. D. Garcia and R. Garcia , “Determination of the Elastic Moduli of a Single Cell Cultured on a Rigid Support by Force Microscopy,” Biophysical Journal 114 (2018): 2923–2932, 10.1016/j.bpj.2018.05.012. 29925028 PMC6026379

[51] S. Maghsoudy‐Louyeh , “Nanomechanical Properties of Biocomposites Using Atomic Force Microscopy—Measurement and Modeling,” The Pennsylvania State University (2011).

[52] R. Steven , T. J. Kubiseski , H. Zheng , et al., “UNC‐73 Activates the Rac GTPase and Is Required for Cell and Growth Cone Migrations in C. Elegans,” Cell 92 (1998): 785–795, 10.1016/S0092-8674(00)81406-3. 9529254

[53] M. Elmi , V. M. Pawar , M. Shaw , D. Wong , H. Y. Zhan , and M. A. Srinivasan , “Determining the Biomechanics of Touch Sensation in C. Elegans,” Scientific Reports 7 (2017): 12329, 10.1038/s41598-017-12190-0. 28951574 PMC5615042

[54] M. Backholm , W. S. Ryu , and K. Dalnoki‐Veress , “Viscoelastic Properties of the Nematode Caenorhabditis Elegans, a Self‐Similar, Shear‐Thinning Worm,” Proceedings of the National Academy of Sciences 110 (2013): 4528–4533, 10.1073/pnas.1219965110. PMC3607018 23460699

[55] S. Brenner , “The Genetics of Caenorhabditis Elegans,” Genetics 77 (1974): 71–94, 10.1093/genetics/77.1.71. 4366476 PMC1213120

[56] T. Stiernagle , “Maintenance of C. Elegans,” WormBook(2006): 1–11. 10.1895/wormbook.1.101.1 PMC4781397 18050451

