# Supplementary material for: Drop‐Shaped Optical Microfiber Enabled Biomechanical Sensor
Source: Adv Sci (Weinh). 2026 May 14:e75673. Online ahead of print. doi: 10.1002/advs.75673 (PMC13335903; doi:10.1002/advs.75673)
Supplement: Supplementary file 1 — Supporting File 1: advs75673‐sup‐0001‐SuppMat.docx. [file ADVS-9999-e75673-s001.docx]

Supporting Information

Drop-Shaped Optical Microfiber Enabled Biomechanical Sensor

*Yan Xu, Xitao Tu, Haochen Jiang, Jun Mo, Xinyue Zhang, Xiaomin Yue*, Limin Tong*, Lei Zhang**


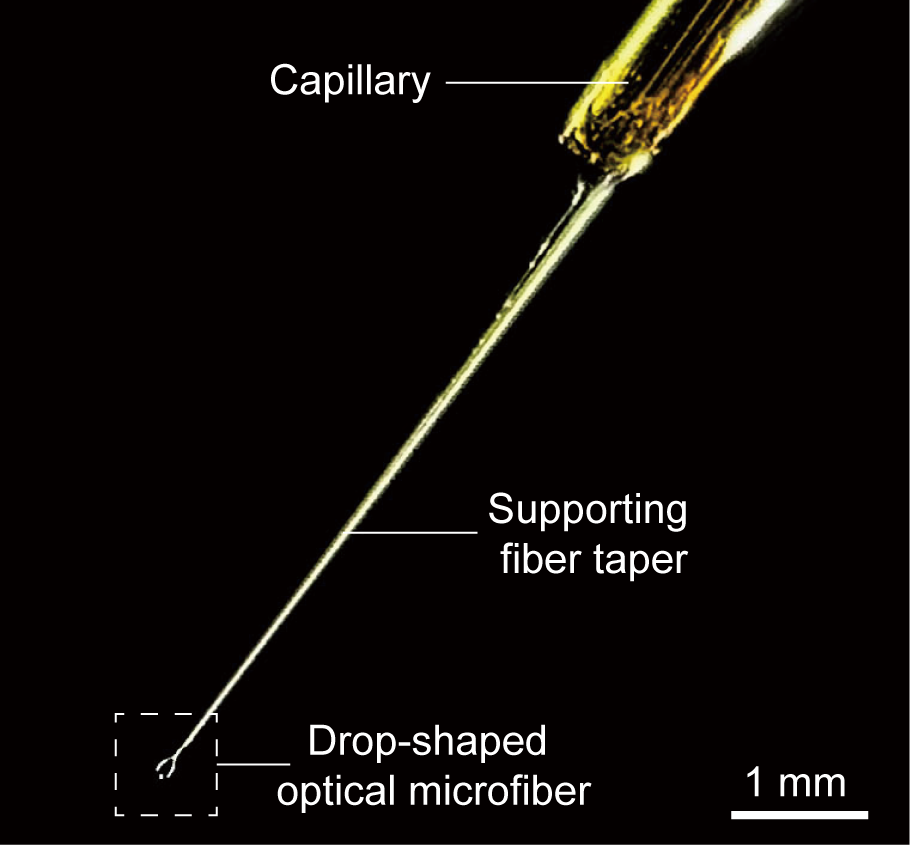


**Figure S1**. Photograph of a drop-shaped optical microfiber sensor.


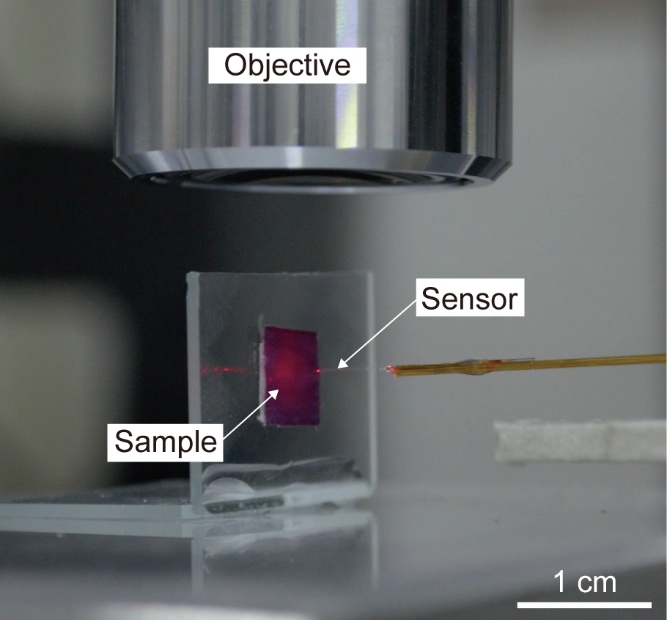


**Figure S2**. Photograph of the experimental setup for indentation test using the drop-shaped optical microfiber sensor.


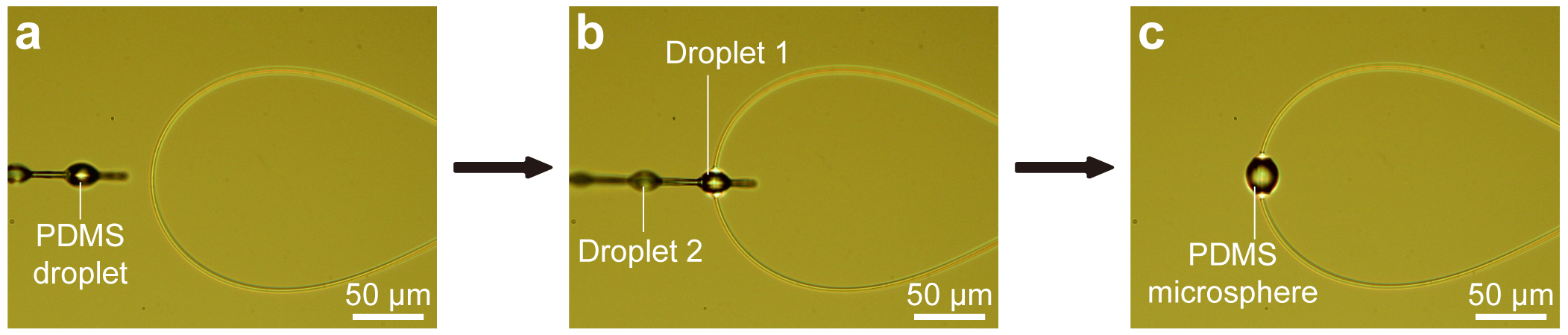


**Figure S3**. Photographs showing the deposition of uncured PDMS droplets onto the tip of a drop-shaped optical microfiber. (a) A fiber taper with a few PDMS droplets approaches the drop-shaped optical microfiber. (b) Droplet 1 is deposited on the drop-shaped optical microfiber via the fiber taper. (c) Two PDMS droplets on the fiber taper are transferred to the drop-shaped optical microfiber forming a PDMS microsphere.


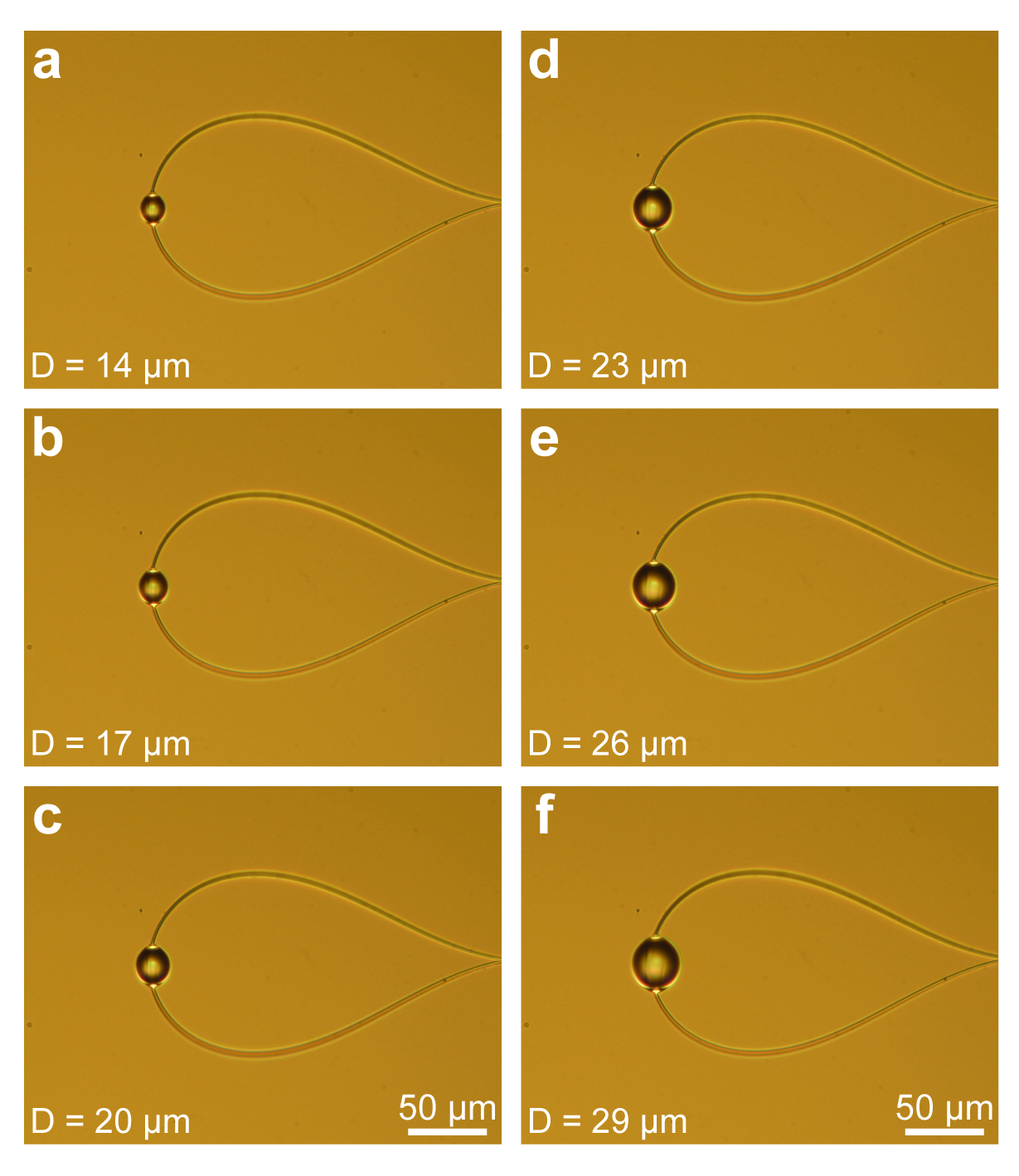


**Figure S4**. Photographs of PDMS microspheres at the drop-shaped optical microfibers. The diameter of each microsphere is labeled in the respective image.


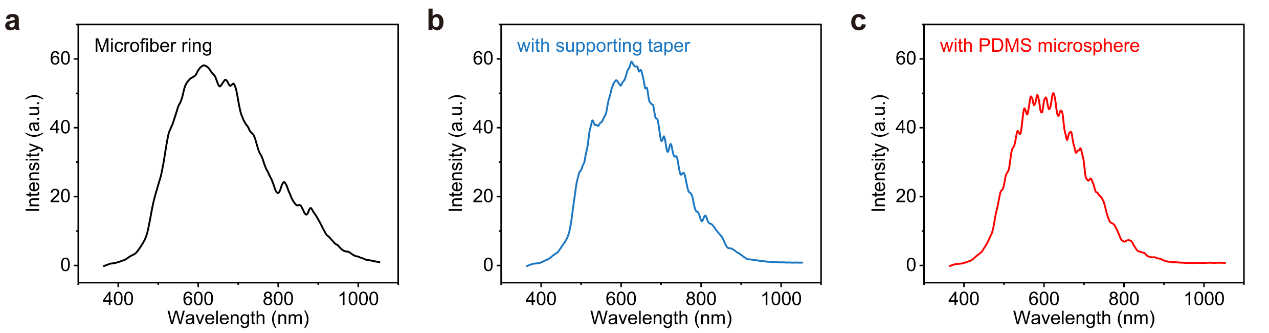


**Figure S5**. Typical transmission spectra of the as-prepared drop-shaped optical microfiber (a), the drop-shaped optical microfiber integrated with a supporting fiber taper (b), and the drop-shaped optical microfiber with a PDMS microsphere (c). The integration time was 1 ms for (a) and (b), and 2 ms for (c).


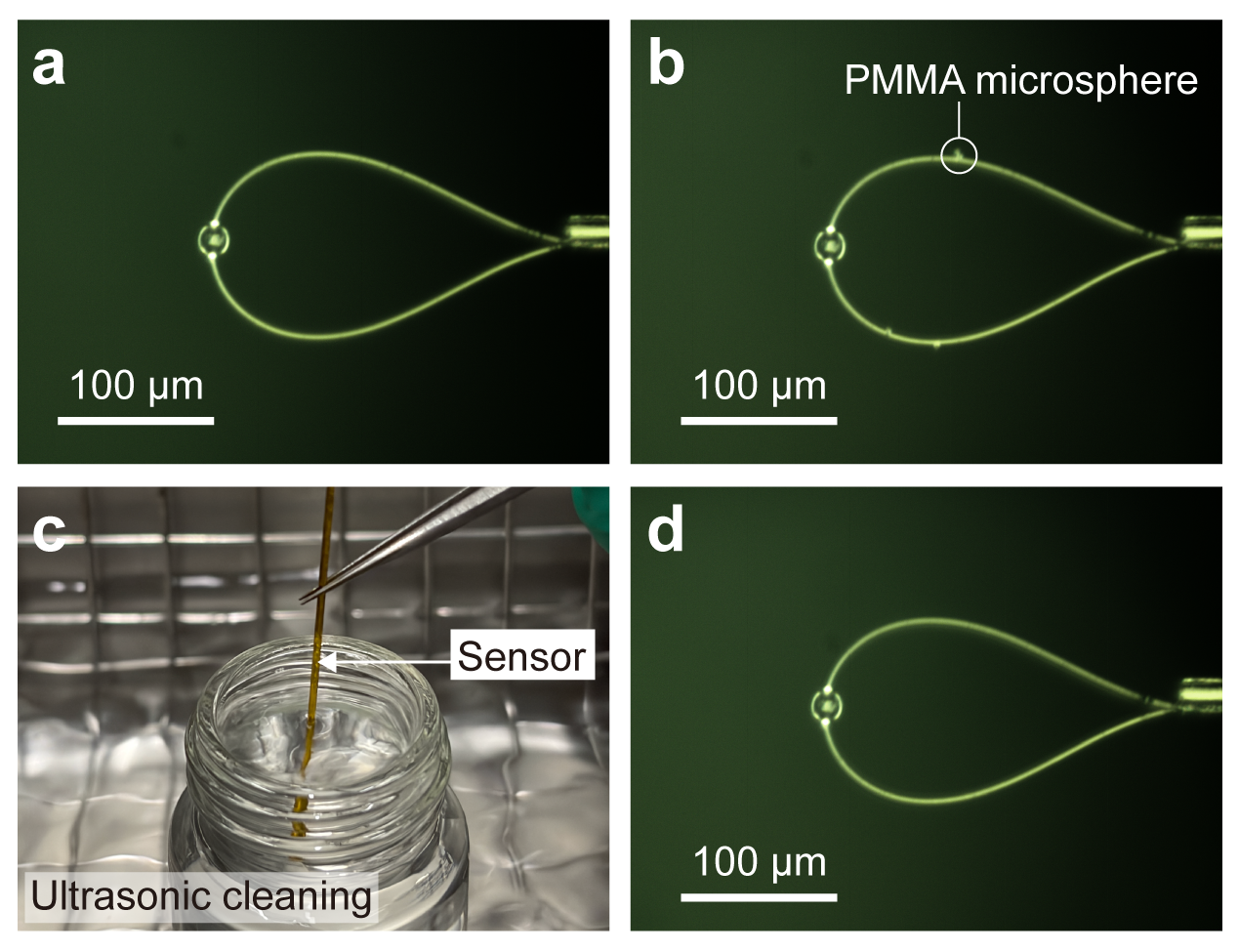


**Figure S6**. Ultrasonic cleaning of the sensor surface. (a) Micrograph of the sensor with a clean surface; (b) Micrograph of the sensor surface with adsorbed PMMA microspheres; (c) Photograph of the sensor placed in an ultrasonic cleaner; (d) Micrograph of the sensor after ultrasonic cleaning, with PMMA microspheres removed.


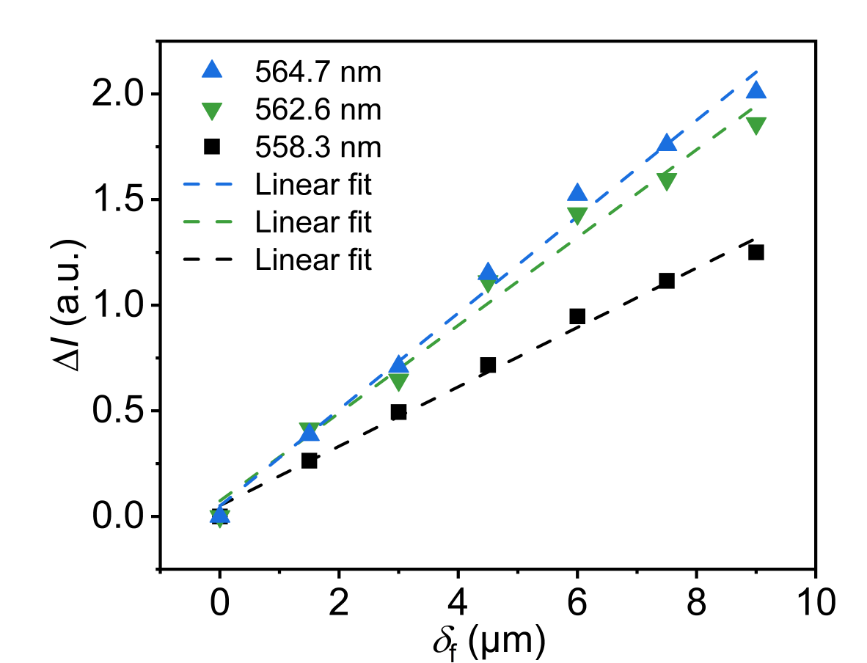


**Figure S7**. Light intensity changes versus the sensor deformation at three different wavelengths, as extracted from Figure 2f.


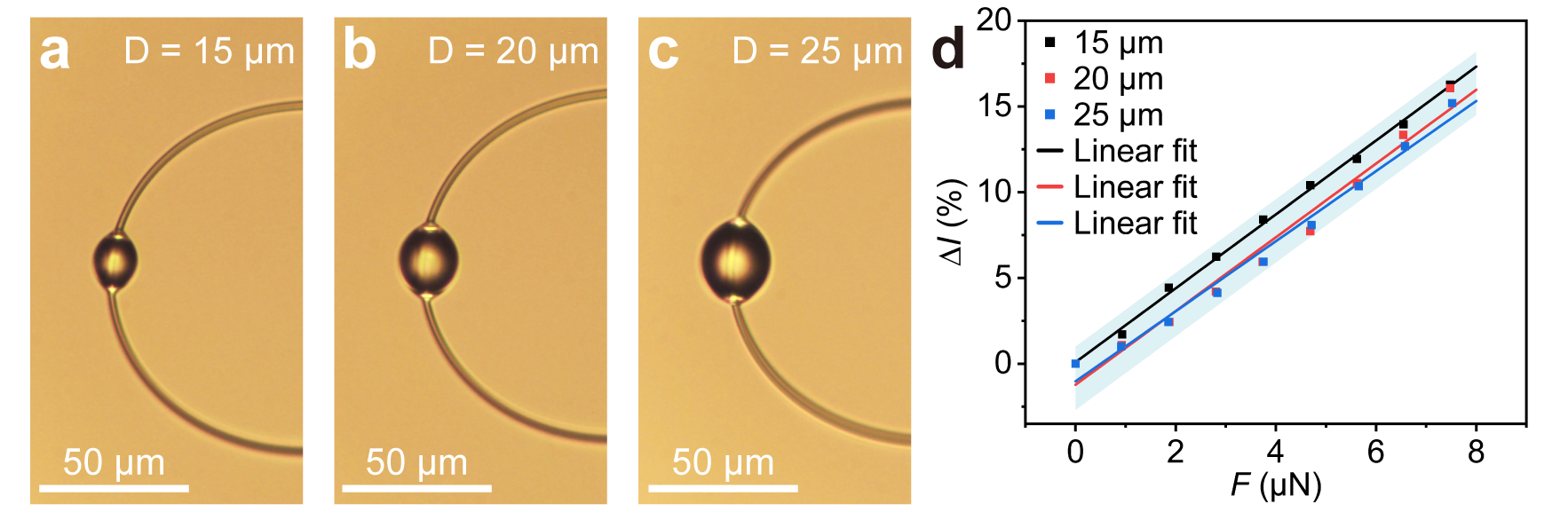


**Figure S8**. Effect of PDMS microsphere diameter on sensing performance. (a-c) Microscopic images of PDMS microspheres with diameters of 15, 20, and 25 μm sequentially fabricated on the same microfiber ring; (d) Linear optical intensity‑pressure responses corresponding to the three PDMS microspheres.


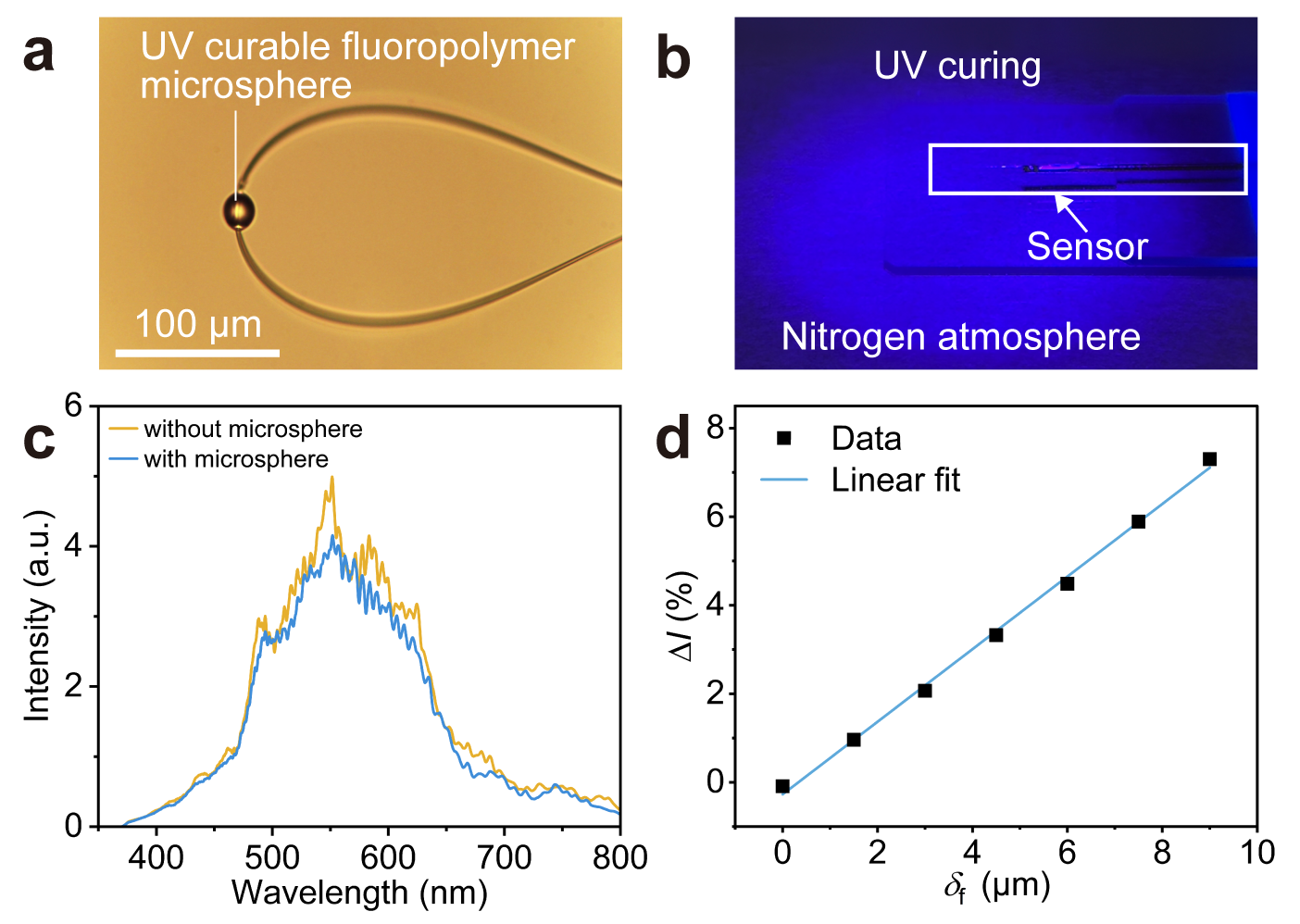


**Figure S9**. Characterization of the microfiber sensor employing UV curable fluoropolymer microspheres instead of PDMS microspheres. (a) Microscopic image of the UV curable fluoropolymer microsphere on the microfiber ring; (b) Photograph of the microsphere cured under UV light in a nitrogen atmosphere; (c) Output spectra of the microfiber ring with and without the UV curable fluoropolymer microsphere; (d) Linear response of the sensor to pressing displacement.


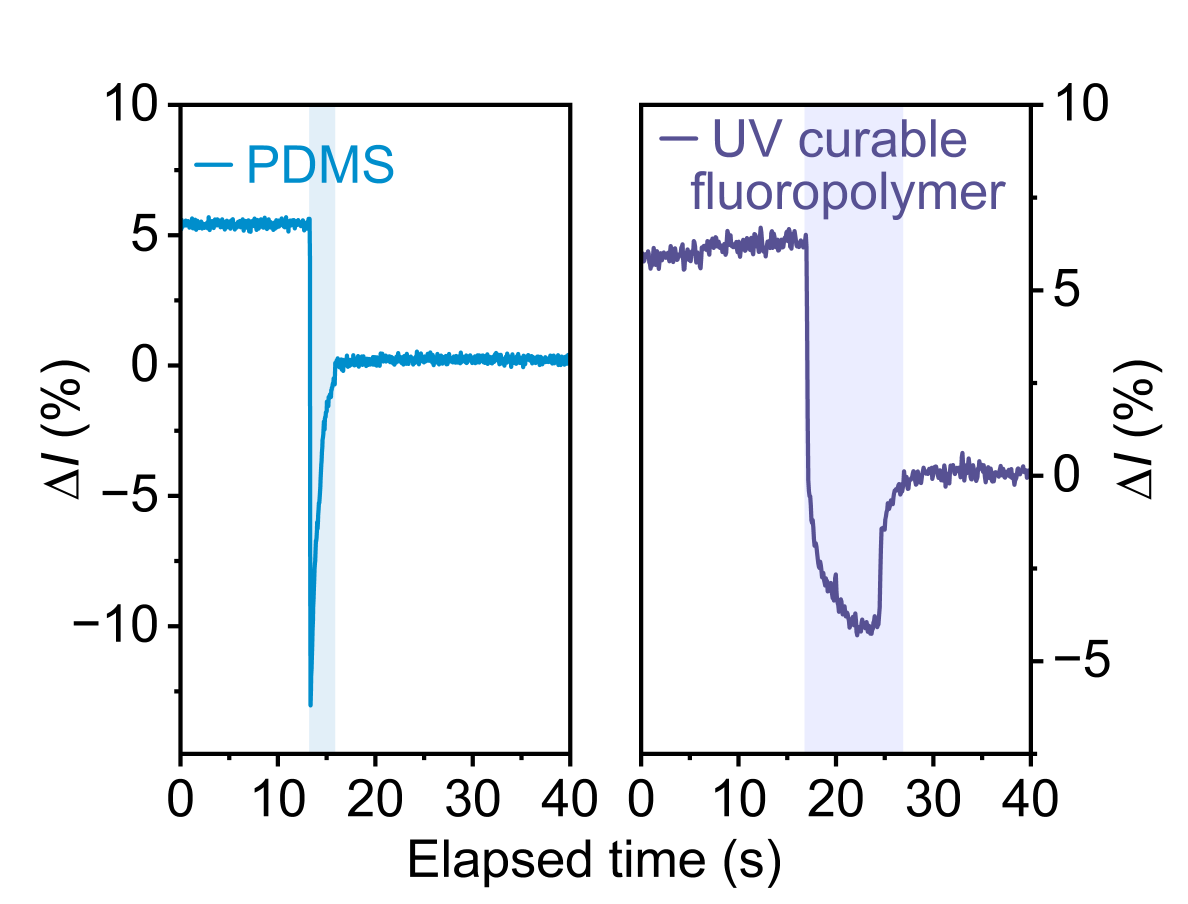


**Figure S10**. Indentation curves of the sensors employing PDMS and UV curable fluoropolymer microspheres, respectively, during detachment from the glass slide after pressing.


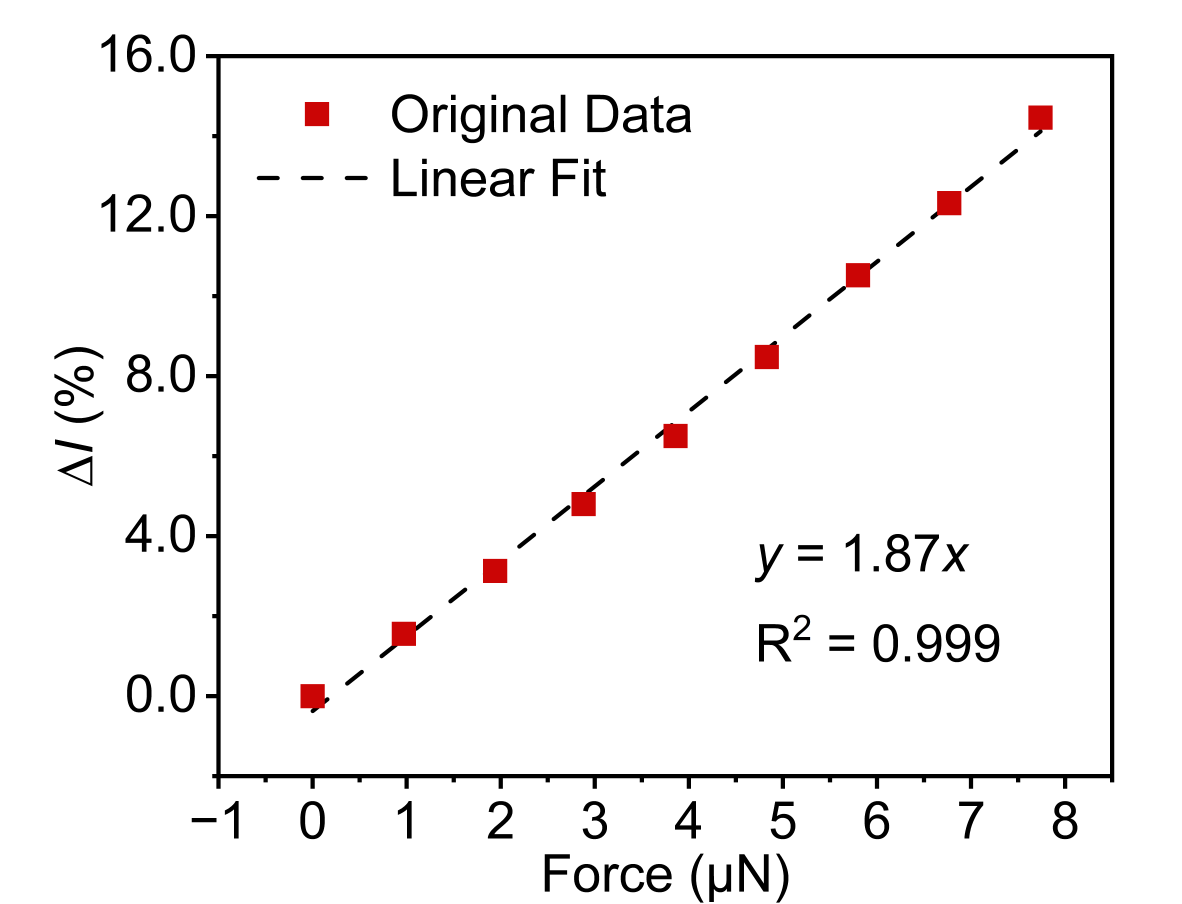


**Figure S11**. Light intensity changes versus applied force for a 120-μm wide drop-shaped optical microfiber operated at the wavelength of 600 nm.


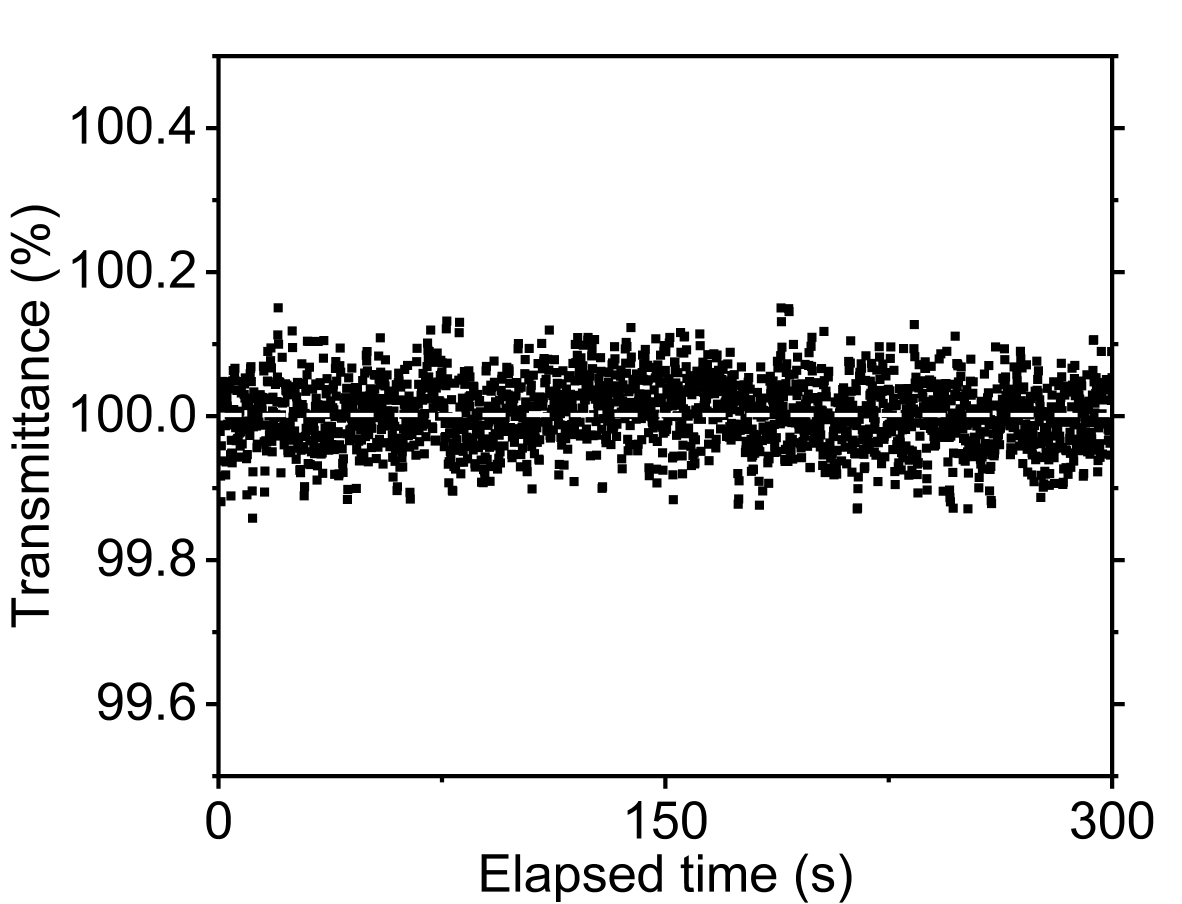


**Figure S12**. Output optical intensity of the sensor under an unloading condition, indicating that the system noise is approximately 0.045%.


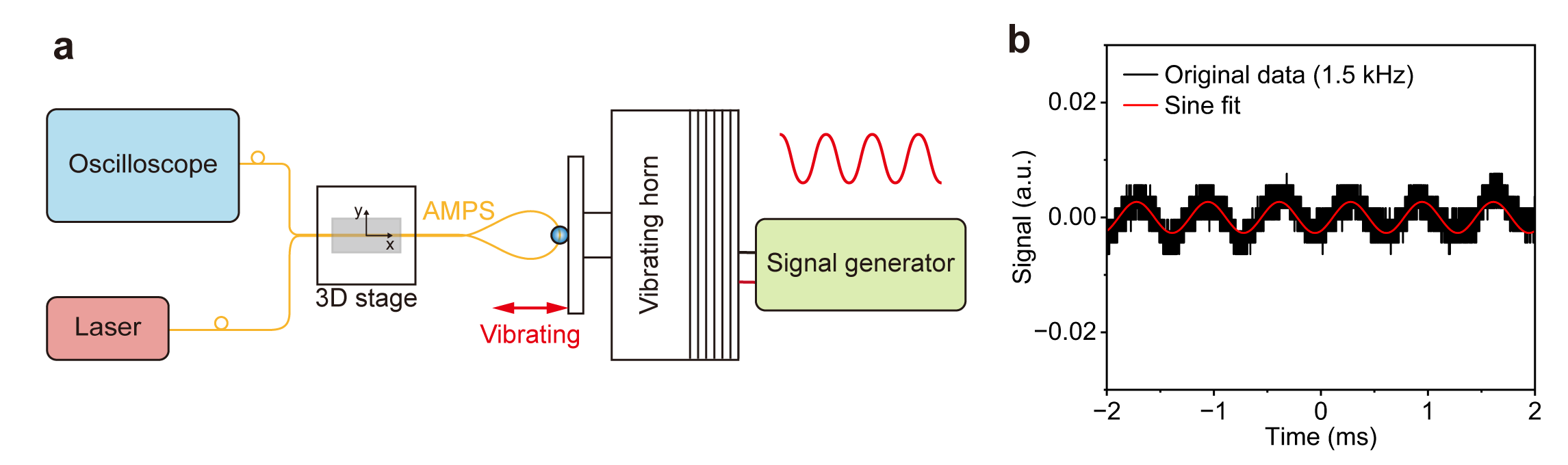


**Figure S13**. Dynamic response of the sensor to high-frequency stimuli. (a) Schematic of the experimental setup for dynamic response test. (b) Response of the sensor to a 1.5- kHz sinusoidal stimuli.


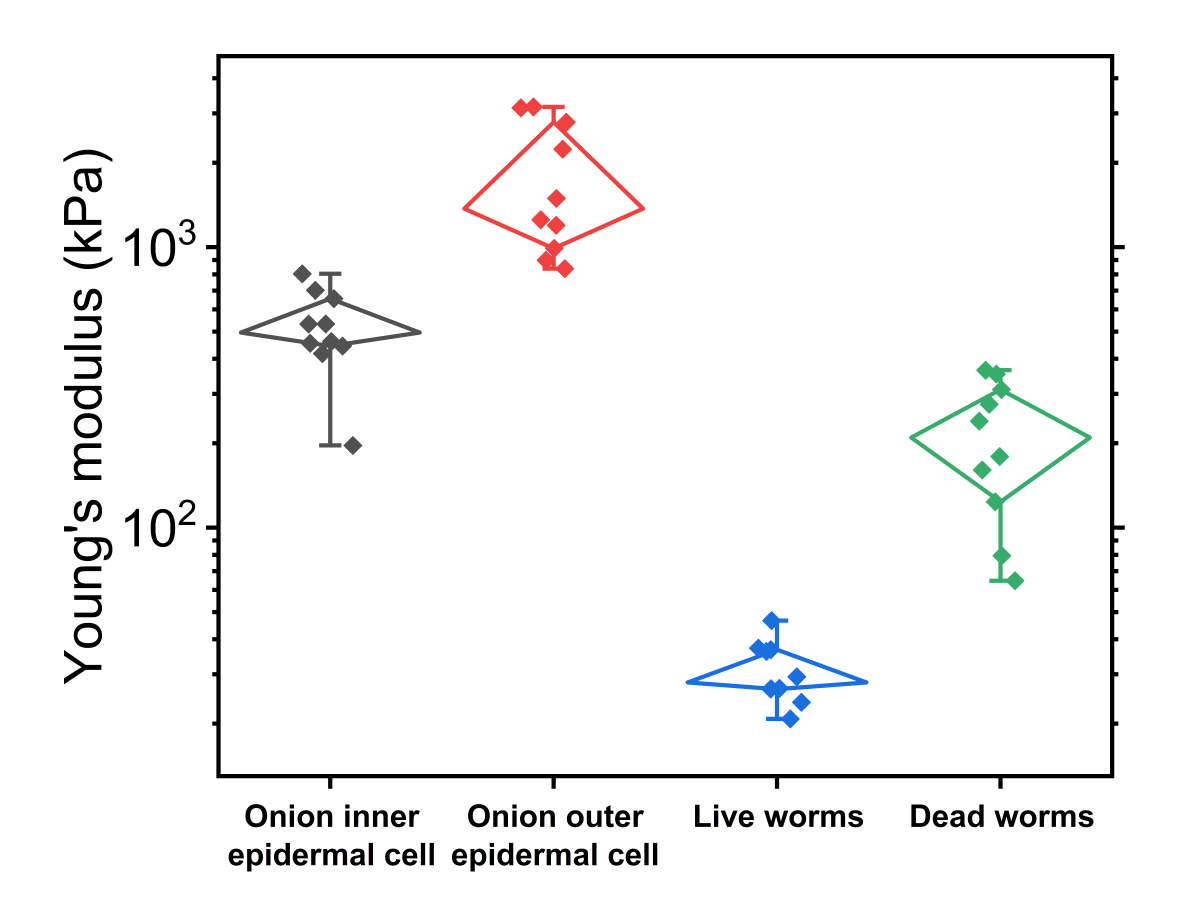


**Figure S14**. Statistical comparison of Young's modulus values of: onion inner epidermal cells; onion outer epidermal cells; live *C. elegans*, and dead *C. elegans*.


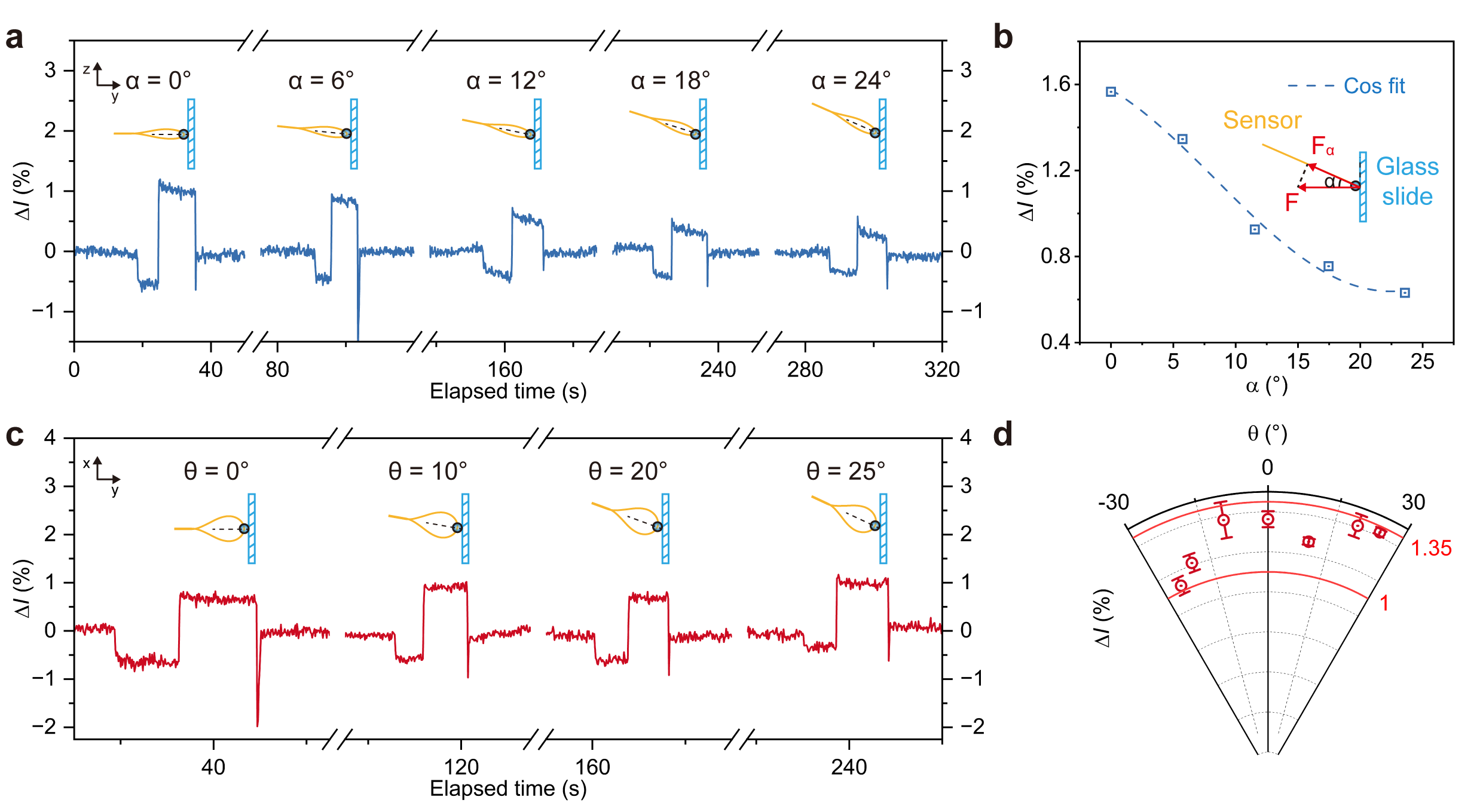


**Figure S15**. Influence of sensor tilt angle on test results. (a) Output light intensity response to pressure when the sensor plane is non-prependicular to the sample surface (title angle α). (b) Cosine relationship between the response signal and the title angle. (c) Output light intensity response to pressure when the sensor plane remains vertical but with a horizontal deflection (deflection angle θ). (d) Polar statistical distribution of response signals at different deflection angles.


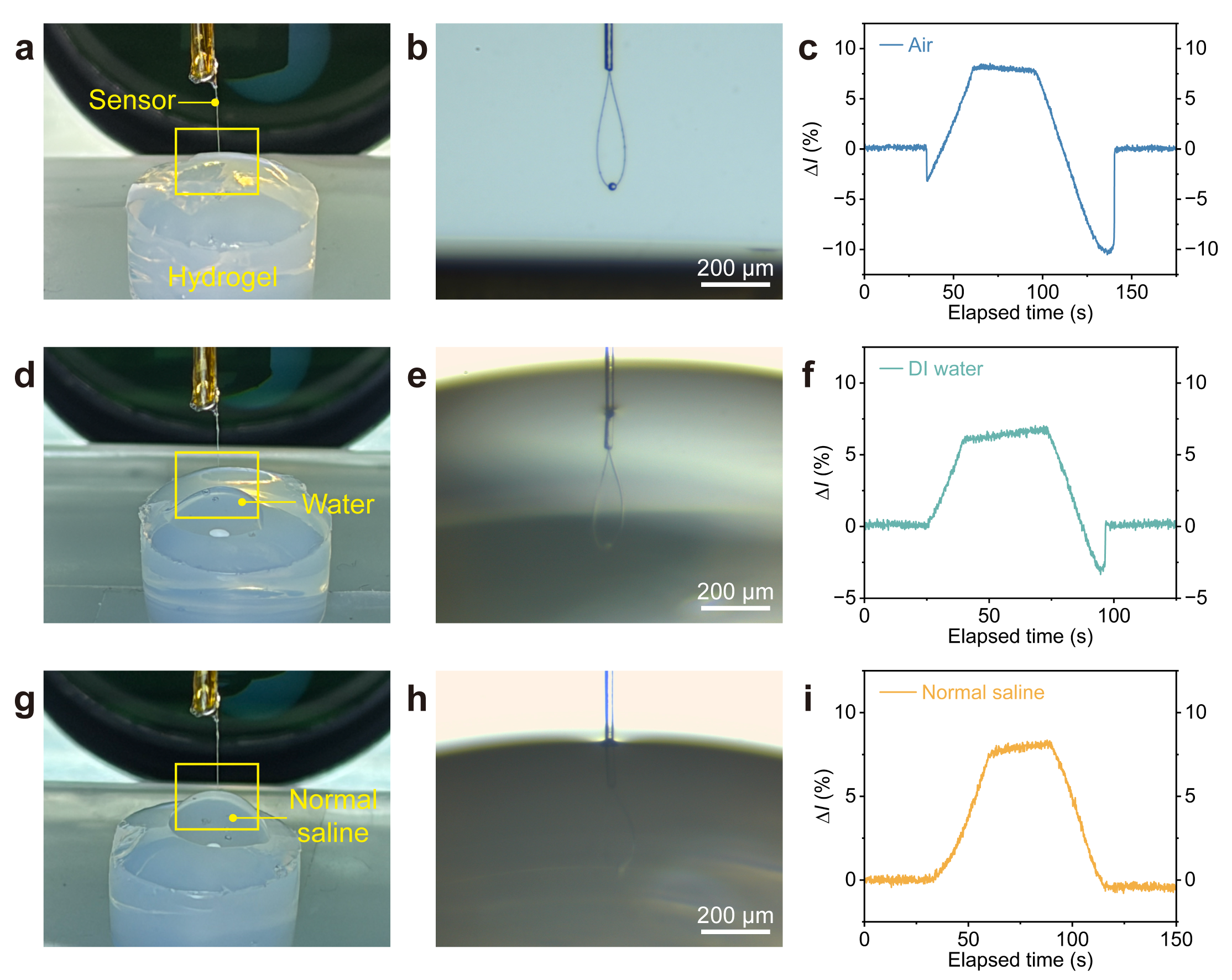


**Figure S16**. Effect of liquid on the hydrogel surface on measurement results. (a-c) Optical photograph (a), microscopic image (b), and corresponding indentation curve (c) obtained when the sensor presses the hydrogel in air; (d-f) Optical photograph (d), microscopic image (e), and corresponding indentation curve (f) obtained when the sensor presses the hydrogel covered with water; (g-i) Optical photograph (g), microscopic image (h), and corresponding indentation curve (i) obtained when the sensor presses the hydrogel covered with normal saline.


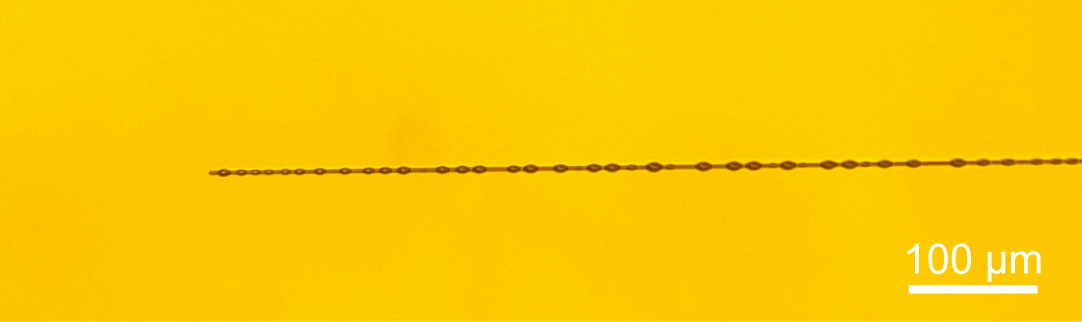


**Figure S17**. Microscopic image of PDMS microspheres attached on the tapered fiber, with droplet volumes ranging from 20 to 200 fL.

**Table S1**. Comparison of Young's modulus measurements via this work and AFM: onion inner epidermal cells; onion outer epidermal cells; live *C. elegans*; and dead *C. elegans*.

| Biological samples | Young's modulus, *E* (kPa) | |
| --- | --- | --- |
|  | **this Work** | **AFM**^[1-3]^ |
| Onion inner epidermal cell | 519.9 ± 170.3 | 200 - 4900 |
| Onion outer epidermal cell | 1799.9 ± 941.1 |  |
| Live worms | 31.1 ± 7.9 | 30 |
| Dead worms | 215.1 ± 109.7 | / |

**Legends for Supplementary Videos**

**Supplementary Video 1 | Biomechanical sensor pressing on a glass slide.** The video demonstrates the sensor approaching, pressing against, and retracting from a glass slide. During pressing, since the slide is non-deformable, all deformation occurs at the sensor side. The drop-shaped optical microfiber expands outward under pressure, correspondingly increasing its bending radius. Upon complete retraction and detachment, the sensor fully recovers to its original state, indicating fully elastic deformation throughout the process. By monitoring the change in output light intensity (Δ*I*) during pressing, the correspondence between Δ*I* and sensor deformation *δ*_f_ can be obtained.

**Supplementary Video 2 | Biomechanical sensor pressing on the free end of a SMF cantilever.** The video demonstrates the process of the sensor pressing against the free end of a single-mode fiber (SMF), causing its deflection, and subsequently retracting. During the pressing progress, both the SMF deflection and the outward expansion of the drop-shaped optical microfiber occur simultaneously, indicating that deformation takes place in both elements during the interaction. The interaction force (*F*) can be calculated from the deflection of the SMF, which is obtained from microscopic images. By monitoring the change in output light intensity (Δ*I*) during pressing, the correspondence between Δ*I* and the external force *F* can be established.

**Supplementary Video 3 | Robustness test via overloading.** The video demonstrates the deformation of the sensor during its interaction with a glass slide under pressing displacements ranging from 0 to 60 μm. Within the initial 0-12 μm after contact, the sensor interacts orthogonally with the slide plane, undergoing only lateral compression in the horizontal direction, during which the output light intensity responds linearly. As the displacement increases to 60 μm, the sensor deflects longitudinally and exhibits significant bending, resulting in a nonlinear optical response. Upon retraction, the sensor returns to its initial orthogonal orientation. A subsequent 0-12 μm pressing reproduces nearly identical lateral compression and linear optical response as in the first cycle. The complete recovery after large bending confirms the high elasticity, and robustness of this optical microfiber-based sensor, while the consistent linear responses across cycles verify its measurement reliability.

**Supplementary References**

1. S. Maghsoudy-Louyeh, “Nanomechanical properties of biocomposites using atomic force microscopy - measurement and modeling,” *The Pennsylvania State University* **2011**, 978-1-303-55405-6.

2. C. L. Essmann, M. Elmi, M. Shaw, G. M. Anand, V. M. Pawar, and M. A. Srinivasan, “In-vivo high resolution AFM topographic imaging of *Caenorhabditis elegans* reveals previously unreported surface structures of cuticle mutants,” *Nanomedicine-Nanotechnology Biology and Medicine* **2017**, *13*, 183-189.

3. M. Elmi, V. M. Pawar, M. Shaw, D. Wong, H. Y. Zhan, and M. A. Srinivasan, “Determining the biomechanics of touch sensation in *C. elegans*,” *Scientific Reports* **2017**, *7*, 12329.
